# Long non-coding RNA DUXAP10 exerts oncogenic properties in osteosarcoma by recruiting HuR to enhance SOX18 mRNA stability

**DOI:** 10.1007/s13577-022-00772-8

**Published:** 2022-09-02

**Authors:** Guantong Wang, Qian Zhang, Qinjue Wang, Jing Wang, Lulu Chen, Qiang Sun, Dengshun Miao

**Affiliations:** 1 Department of Orthopedics, Nanjing First Hospital, Nanjing Medical University, Nanjing, China; 2 State Key Laboratory of Reproductive Medicine, Research Center for Bone and Stem Cells, Key Laboratory for Aging and Disease, Nanjing Medical University, Nanjing, China

**Keywords:** Osteosarcoma, DUXAP10, SOX18, HuR, LncRNAs

## Abstract

Recent studies have demonstrated that several long non-coding RNAs (lncRNAs) play an important role in the occurrence and development of osteosarcoma (OS). However, more lncRNAs and their mechanisms in regulating growth and migration of OS cells remain to be investigated. In this study, we identified an lncRNA called DUXAP10 by analysis of GEO data, which was significantly up-regulated in OS tissues and cell lines. Experiments in vitro revealed that lncRNA DUXAP10 promoted proliferation, migration, and invasion of OS cells and inhibited their apoptosis. We also demonstrated that DUXAP10 promoted the formation and growth of OS by tumor formation assay. Furthermore, SOX18 was identified as a critical downstream target of DUXAP10 by transcriptome RNA-seq. Mechanistically, DUXAP10 mainly localized in cytoplasm and could specifically bind to HuR to increase the stability of SOX18 mRNA. Meanwhile, SOX18 knockdown largely reversed increased proliferation of OS cells induced by DUXAP10 overexpression. Findings from this study indicate that lncRNA DUXAP10 can act as an oncogene in osteosarcoma by binding HuR to up-regulate the expression of SOX18 at a post-transcriptional level, which may provide a new target for OS clinical diagnosis and treatment.

**Supplementary Information:**

The online version contains supplementary material available at 10.1007/s13577-022-00772-8.

## Introduction

Osteosarcoma (OS) is the most common primary malignant bone tumor in children and adolescents, mainly located in the metaphysis of long bones [1–3]. It has a high degree of malignancy and poor prognosis. Besides, lung metastasis often occurs in the early stage of OS. With the improvement of therapeutic techniques, such as chemotherapy, radiotherapy, and extensive tumor resection, the 5-year survival rate of OS patients is significantly improved [4–6]. However, currently, due to the lack of diagnostic and precision treatment techniques, half of the OS patients still have lung metastases, and only a small percentage of patients can be completely cured [7, 8]. Therefore, it is necessary to find new osteosarcoma biomarkers and therapeutic targets.

Long non-coding RNAs (lncRNAs) are a class of ncRNAs with a transcript length > 200 nt. Different from small non-coding RNAs, they can regulate the expression of target genes at epigenetics, transcription, and post-transcriptional levels, forming a complex network of gene expression and regulation to affect tumorigenesis and metastasis [9–11]. There have been some reports about lncRNAs in osteosarcoma, such as: lncRNA casc2 promotes the growth and invasion of OS by regulating miR-181a [12]; lncRNA ANRIL regulates caspase-3, Bcl-2, MTA1, and TIMP-2 to promote the progression of OS [13]; LINC01116 regulates miR-520a-3p through the Jak–stat pathway to promote OS proliferation and invasion [14]. Obviously, lncRNAs play an important role in the occurrence and development of osteosarcoma. Therefore, it is important to explore more OS-related lncRNAs and their regulatory mechanisms, which is useful for understanding the biological laws of the development, proliferation, and migration of OS, providing experimental and theoretical basis for clinical diagnosis and treatment.

We first analyzed the OS case–control chip in the GEO database (Gene Expression Omnibus), and found that lncRNA double homeobox A pseudogene 10 (DUXAP10) was significantly up-regulated in 19 OS tissues. DUXAP10 is 2398 nt length and located in 14q11.2. Previous studies have shown that DUXAP10 could promote tumor progression in non-small cell lung cancer [15], colorectal cancer [16], gastric cancer [17], pancreatic cancer [18], esophageal squamous cell carcinoma [19], and bladder cancer [20]. However, its role in OS has not been reported. Thus, in this study, we determined the effects of DUXAP10 on OS proliferation and migration in cells, and demonstrated the underlying mechanism of DUXAP10 in the development of OS.

## Materials and methods

### lncRNA expression from GEO datasets

OS gene expression was obtained from the GEO database (GSE28424 data set). After downloading the original file from the GEO database, we normalized it using Robust Multichip Average (RMA) and reannotated it for lncRNA by blast + 2.2.30. Compared to normal tissues, lncRNAs differentially expressed in osteosarcoma tissues were selected.

### Clinical samples

The OS tissues used in this study were obtained from OS patients who underwent tumor resection in the Nanjing First Hospital. All patients had been histopathologically diagnosed and had no local or systemic treatment before surgery. Normal bone tissues were obtained from the discarded bone tissues of male patients with severed fingers undergoing stump trimming. All samples were frozen in liquid nitrogen immediately and stored at − 80 °C. This study was approved by the Ethics Committee of Nanjing First Hospital, and all patients have signed informed consent.

### Cell lines and cell culture

The OS cell lines (U2OS, SAOS2, HOS, and MG63) and human osteoblast cell hFOB1.19 were purchased from the Chinese Academy of Medical Sciences. The cells were cultured in Dulbecco’s Modified Eagle Medium (DMEM) (Gibco, USA) containing 1% penicillin streptomycin (Gibco, USA) and 10% Fetal Bovine Serum (FBS, BI) at 37 °C under humidified atmosphere with 5% CO_2_.

### RNA extraction and qPCR assays

Total RNA was extracted from tissues or cells using Trizol reagent (Invitrogen, USA) and the RNA concentration was determined by NanoDrop ND-2000 (Thermo Fisher Scientific, Wilmington, DE, USA). Then, 1 μg RNA was reverse-transcribed to 20 μl cDNA using random primers under standard conditions for the PrimeScript RT reagent Kit (TaKaRa, Dalian, China). Quantitative real-time PCR (qPCR) was carried out on Applied Biosystems 7500 Real-Time PCR System (Applied Biosystems) using SYBR Premix Ex Taq (TaKaRa, Dalian, China), and GADPH was served as an internal control. At last, the CT value was analyzed using the 2^−∆∆ct^ method. The experiment was repeated three times independently. All primers used in this study were included in supplemental data (Table S1). All reactions were executed in triplicate.

### Cell transfection

According to the manufacturer’s instructions, cells in 6-well plates (Corning Incorporated) were transfected with siRNA using lip2000 (Invitrogen, Carlsbad, CA, USA) or X-tremeGENE (Roche, Shanghai, China). The transfected cells were cultured in Opti-MEM medium (Gibco, USA) for 6 h and then replaced with normal medium. Cells were harvested 48 h later for qPCR or Western blot analysis. All siRNA sequences used in this study were included in supplementary data (Table S2).

### MTT assay

After transfected with siRNA for 24 h, cells were seeded in 96-well plates (Corning Incorporated) at 200 μl per well at 1.5 × 10^6^ cells/ml. MTT was added after 0, 24, 48, 72, and 96 h, respectively, and the optical density at 490 nm (OD490nm) was measured by the microplate reader. The experiment was repeated three times independently.

### Cell colony formation assay

While performing the MTT assay, we seeded 1500 cells into 6-well plates. One week later, the cells were fixed with methanol and stained with 0.1% crystal violet. The experiment was repeated three times independently.

### EdU assay

After transfected with siRNA for 24 h, the cells were subjected to EdU staining (Ribobio, Guangzhou, China) according to the manufacturer’s instructions. Then, images of EdU-positive cells were captured under a fluorescence microscope (Olympus, Tokyo, Japan, 200×). The experiment was repeated three times independently.

### Cell migration and invasion assays

After transfected with siRNA for 24 h, we seeded 60,000 cells in a 24-well Transwell chamber (Corning Incorporated) with Matrigel (BD Biosciences) for cell invasion assay and 40,000 cells in a 24-well Transwell chamber without Matrigel for cell migration assay. After cultured for 24 h, the cells in the upper chamber were removed with a cotton swab, and the cells on the surface of the inferior membrane were fixed and stained with 0.1% crystal violet. For cell invasion assay, we collected cells 48 h after planting. Cells passing through the membrane were counted under an inverted microscope (Olympus, Tokyo, Japan, 100 ×). The experiment was repeated three times independently.

### Stable expression cell line construction and tumor formation assay

U2OS cells were infected with lentiviral-packed sh-DUXAP10 (GENE, Shanghai) and stable-infected cells were screened with medium containing puromycin (Sigma, 2 μg/ml). The 4-week-old male nude mice used in the experiment were purchased from Model Animal Research Center of Nanjing University and were raised in standard pathogen-free conditions. U2OS cells stably transfected with sh-DUXAP10 or empty vector were digested and centrifuged, and resuspended in PBS at a concentration of 2 × 10^7^ cells/ml. Then, 0.1 ml cells were subcutaneously injected into each nude mouse. Tumor size was measured every 3 days after tumor appearance. Three weeks after the injection, the tumor was taken out from sacrificed mice, and the size and weight of the tumor were measured. Tumor tissues were used for qRT-PCR analysis of DUXAP10 expression levels and immunostaining analysis of Ki-67 protein expression (Olympus, Tokyo, Japan, 400×).

### Cell cycle and apoptosis assay

After transfected with siRNA for 48 h, the cells were digested, centrifuged, and washed with ice-cold PBS. Then, cells were stained with propidium oxide using the Cycle TEST PLUS DNA Reagent Kit (BD Biosciences) following the manufacturer’s protocol and analyzed by FACScan according to the manufacturer's instructions. For apoptosis analysis, cells transfected for 48 h were harvested and treated with Annexin V-FITC and PI (BD Biosciences, USA) in the dark according to the manufacturer's instructions.

### RNA-seq analysis

The RNA-seq were conducted in the Novogene (Beijing, China), and the mRNA-seq library was obtained according to standard protocols (Illumina, San Diego, CA, USA). In brief, total RNA from si-NC or si-DUXAP10-1#-transfected U2OS cells was isolated as described above and mRNA was purified using Dynabeads Oligo (dT) (Invitrogen Dynal) and reverse-transcribed into cDNA, which was then fragmented by nebulization to establish the mRNA-seq library. Then, the differentially expressed genes were screened using the RSEM software and the differential gene expression patterns were analyzed by cluster analysis. GO annotation analysis of the distribution of gene functions was performed using WEGO software (http://wego.genomics.org.cn). Also, pathway enrichment analysis of the interaction between genes was analyzed by the KEGG database. The sequencing results were included in supplementary data (Table S3).

### Subcellular fractionation

The separation of nuclear and cytosolic fractions was performed using the PARIS Kit (Life Technologies, Carlsbad, CA, USA) according to the manufacturer’s instructions.

### RNA immunoprecipitation (RIP) assay

RIP experiments were carried using a Magna RIP RNA-Binding Protein Immunoprecipitation Kit (Millipore) according to the manufacturer’s protocol. Antibodies for RIP assays against HuR were purchased from Millipore.

### Western blot

In Western blot analysis, protein was extracted with RIPA radio immunoprecipitation assay (RIPA) protein extraction reagent (Beyotime) containing phenylmethanesulfonyl fluoride (PMSF), and the protein concentrations were detected using a bicinchoninic acid (BCA) protein assay kit (Thermo Fisher Scientific). Proteins were separated by sulfate-polyacrylamide gel electrophoresis (SDS-PAGE) and transferred onto a polyvinylidene difluoride membrane (Millipore, Billerica, MA, USA). The membranes were blocked in tris-buffered saline Tween-20 (TBST) with 5% nonfat milk for 2 h at room temperature and then incubated with primary antibodies at 4 °C overnight. The next day, membranes were washed 3 times with 1× TBST buffer and then incubated with the corresponding secondary antibody at 37 °C for 1 h. After being washed 3 times, the membranes were visualized using an ECL Plus Detection Kit (Pierce, Rock- ford, IL, USA). GAPDH served as the internal control. All antibodies used in this study were included in supplemental data (Table S4).

### HE staining

Tumors from mice model were immersed in 4% paraformaldehyde for 4 h, and transferred to 70% ethanol. Tumors were placed in processing cassettes, dehydrated through a serial alcohol gradient, and embedded in paraffin wax blocks. Before immunostaining, tumors were dewaxed in xylene, rehydrated through decreasing concentrations of ethanol, and washed in PBS. And then stained with hematoxylin and eosin (H&E). After staining, sections were dehydrated through increasing concentrations of ethanol and xylene.

### Ki67, p-Akt, and cleaved Caspase3 staining

For tumors from mice model, Ki67, p-Akt, and Cleaved Caspase3 were detected by standard immunohistochemistry protocols. Slides were deparaffinized, hydrated, and boiled in EDTA buffer (pH 9.0) for 2 min for antigen retrieval. After treated with 3% H2O2 for 25 min, slides were blocked with 3% BSA for 30 min, and then incubated with a rabbit antibody (1:100, GB13030-2, Servicebio, China) overnight at 4 °C, followed by an incubation with HRP-conjugated secondary antibody (Servicebio) for 50 min. Then, the signal was developed by in DAB (brown) solution for 5 min and the nuclear was counterstained with hematoxylin (blue).

### Statistical analysis

All data were derived from at least 3 independent experiments and expressed as mean ± SEM. Differences between groups were evaluated with two-tailed Student’s *t* test using GraphPad Prism software 6.0 software. *p* < 0.05 was considered statistically significant.

## Results

### DUXAP10 is significantly up-regulated in OS tissues and cells

**Fig. 1:**
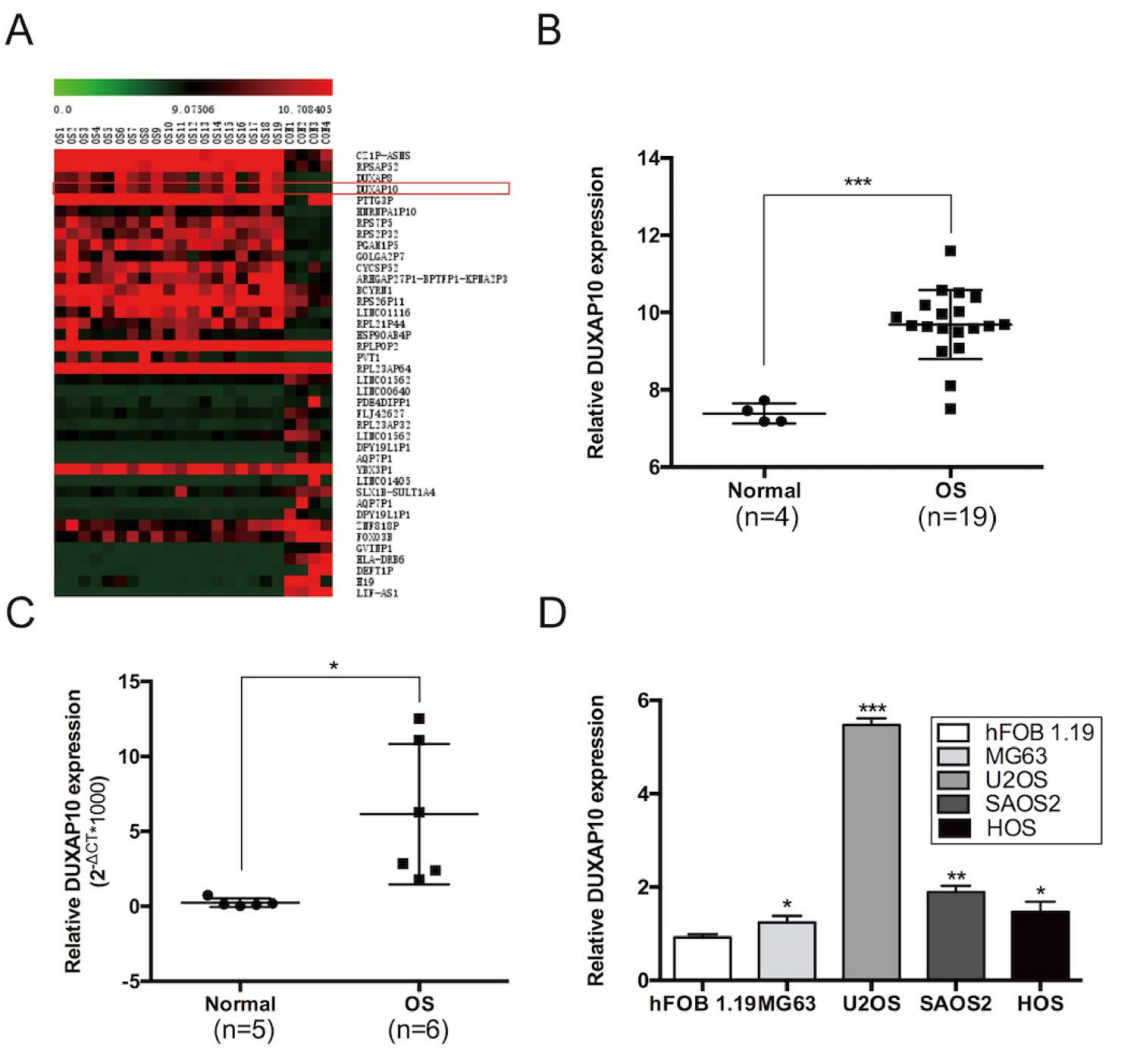
DUXAP10 is significantly up-regulated in OS tissues and cells. **A** The expression profile of lncRNAs in GSE28424 data (https://www.ncbi.nlm.nih.gov/gds). **B** Expression of DUXAP10 in OS and normal bone tissues from database. **C** Expression of DUXAP10 in OS and normal bone tissues we collected. **D** Expression of DUXAP10 in four OS cell lines and a human osteoblast cell line. Data are the means ± SEM of three experiments. *P < 0.05, **P < 0.01, ***P < 0.001

We performed a biological information analysis on an OS case–control chip data (GSE28424) from GEO database (https://www.ncbi.nlm.nih.gov/gds) and found many differentially expressed lncRNAs. Among these lncRNAs, DUXAP10 was significantly up-regulated in OS tissues (Fig. 1A, B). Then, we also found the high expression of DUXAP10 in OS tissues we collected by qRT-PCR assay (Fig. 1C). At the same time, we demonstrated that the expression levels of DUXAP10 were significantly up-regulated in OS cell lines, including MG63, U2OS, SAOS2, and HOS (Fig. 1D). Because DUXAP10 was up-regulated more highly in U2OS and SAOS2 cells, these two cell lines were used for subsequent experiments. These results indicated that DUXAP10 was highly expressed in OS tissues and cells, suggesting that it may be an important lncRNA for OS.

### DUXAP10 promotes proliferation of OS cells in vitro

**Fig. 2:**
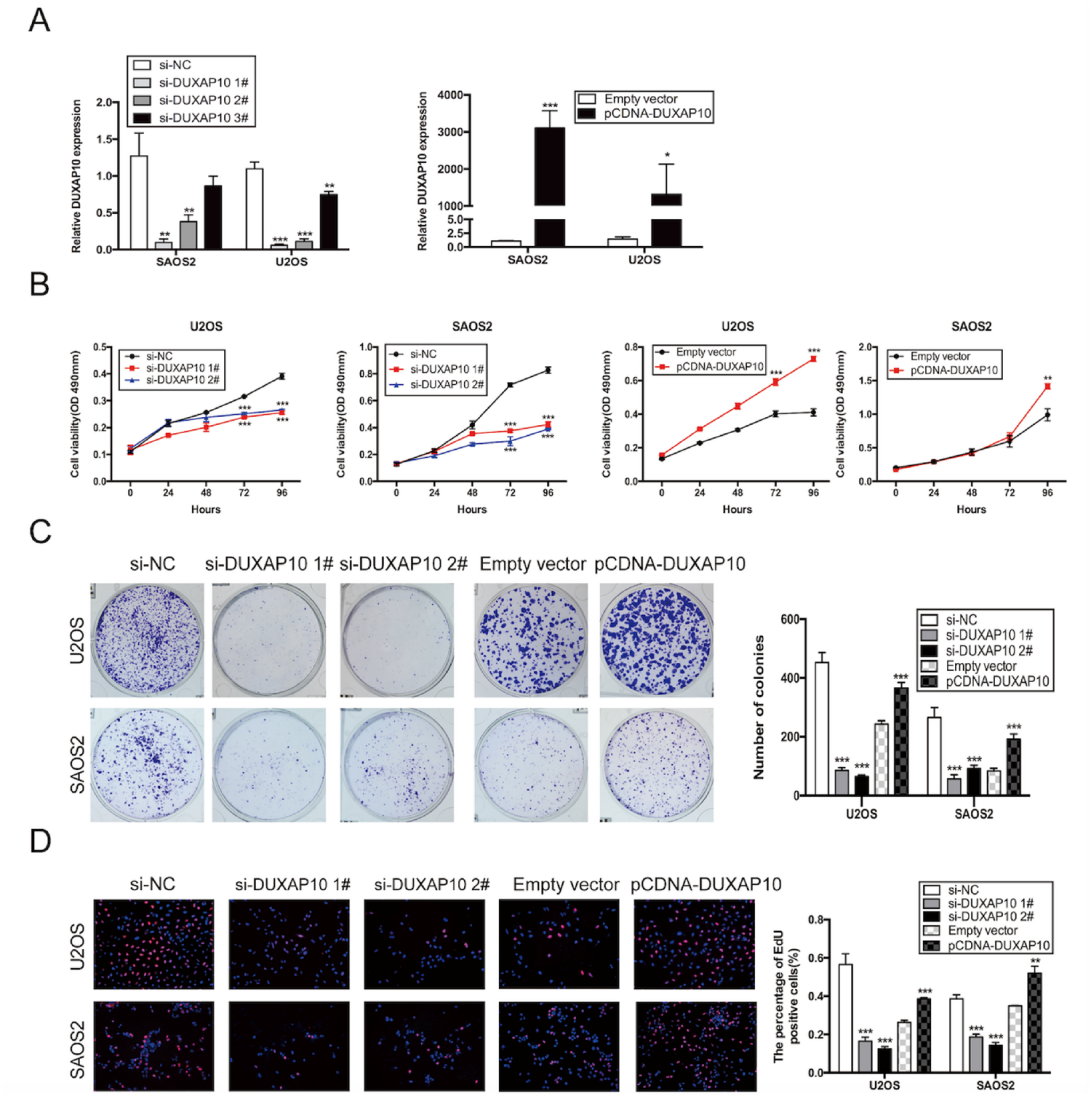
DUXAP10 promotes OS cell proliferation in vitro. **A** Relative DUXAP10 expression in DUXAP10 knockdown or overexpression U2OS and SAOS2 cells. **B** MTT assay was used to detect the effect of DUXAP10 on OS cell proliferation activity. **C** Colony formation assay was used to detect the effect of DUXAP10 on OS cell plate colony formation ability. **D** EdU assay was used to detect the effect of DUXAP10 on OS cell proliferation activity. Data are the means ± SEM of three experiments. *P < 0.05, **P < 0.01, ***P < 0.001

To explore the biological function of DUXAP10 in OS cells, we first designed siRNA and overexpression plasmids to specifically knockdown and overexpress DUXAP10 in U2OS and SAOS2 cells (Fig. 2A). Si-DUXAP10 1# and 2#, with higher knockdown efficiency, were chosen for subsequent experiments. To investigate the effect of DUXAP10 on OS cell proliferation, we knocked down or overexpressed DUXAP10 in U2OS and SAOS2 cells, and used MTT, colony formation assay, and EdU assay to detect the change of OS cell proliferation. The results showed that the cell proliferation activity, plate colony formation ability, and the percentage of EdU-positive cells were significantly reduced in the DUXAP10 knockdown U2OS and SAOS2 cells compared with the control cells, while these parameters were significantly increased in the DUXAP10 overexpressing U2OS and SAOS2 cells (Fig. 2B–D). These results demonstrated that DUXAP10 can promote OS cell proliferation.

### DUXAP10 promotes OS cell migration and invasion, inhibits OS cell apoptosis, and affects cell cycle progression in vitro

**Fig. 3:**
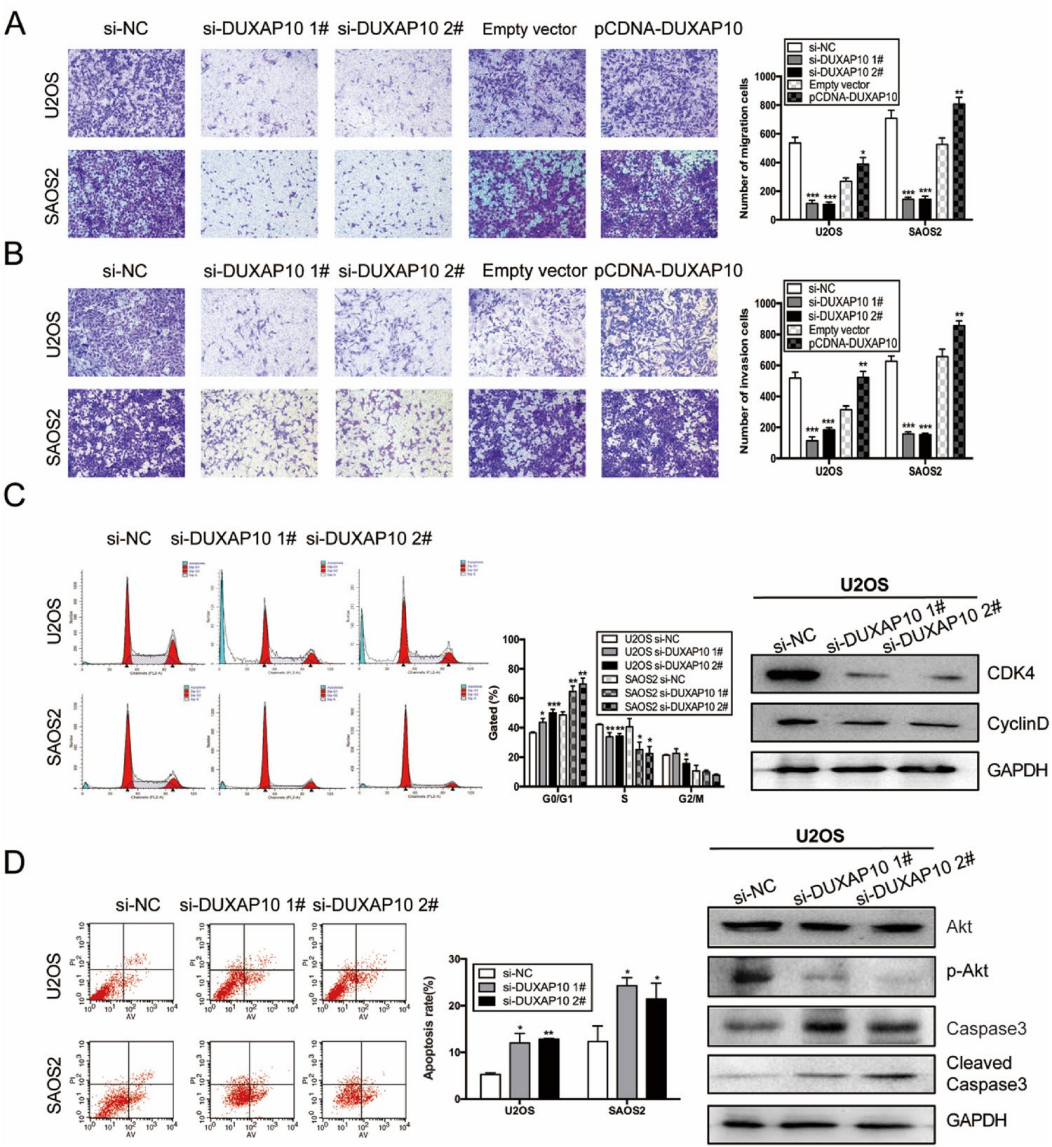
DUXAP10 promotes OS cell migration and invasion, inhibits cell apoptosis, and affects cell cycle progression in vitro. **A** Transwell chamber assay was used to detect the effect of DUXAP10 on OS cell migration ability. **B** Transwell chamber with Matrigel matrix assay was used to detect the effect of DUXAP10 on OS cell invasion ability. **C** Flow cytometry assay was used to detect the effect of DUXAP10 on OS cell cycle; Western blot assay was used to detect the change of cell cycle-regulated proteins expression in DUXAP10 knockdown U2OS cells. **D** Flow cytometry assay was used to detect the effect of DUXAP10 on OS cells apoptosis; Western blot assay was used to detect the change of cell apoptosis-regulated proteins expression in DUXAP10 knockdown U2OS cells. Data are the means ± SEM of three experiments. *P < 0.05, **P < 0.01, ***P < 0.001

To explore the effect of DUXAP10 on OS cell migration and invasion, U2OS and SAOS2 cells with knockdown or overexpression of DUXAP10 were used for Transwell chamber (migration) cultures or Transwell chamber cultures with Matrigel matrix (invasion). The results showed that the number of cells passing through the basement membrane or Matrigel was significantly reduced in DUXAP10 knockdown U2OS and SAOS2 cells, but it was significantly increased in DUXAP10 overexpressing cells (Fig. 3A, B). Since the cell cycle of tumor cells is in a rapidly dividing state, to examine whether DUXAP10 affects the proliferation of OS cells by regulating cell cycle, we used flow cytometry assay to detect cell cycle changes in DUXAP10 knockdown U2OS and SAOS2 cells. The expression levels of cell cycle-regulated proteins were detected in the DUXAP10 knockdown cells by Western blot. The results showed that the cells in G0/G1 phase were significantly increased, whereas the cells in S phase were decreased and Cyclin D and Cyclin-dependent kinase 4 (CDK4) were significantly down-regulated in the DUXAP10 knockdown cells compared with the control cells (Fig. 3C). We also examined the effect of DUXAP10 on OS cell apoptosis and apoptosis-related protein expression levels. The results showed that the percentage of apoptotic tumor cells was significantly increased in DUXAP10 knockdown cells, which showed the down-regulation of the phosphorylated protein kinase B (p-Akt) and the up-regulation of Cleaved Caspase3 expression levels (Fig. 3D). These results demonstrated that DUXAP10 can promote OS cell migration and invasion, inhibit OS cell apoptosis, and affect their cell cycle progression in vitro.

### Knockdown of DUXAP10 inhibits OS cell tumorigenesis in vivo

**Fig. 4:**
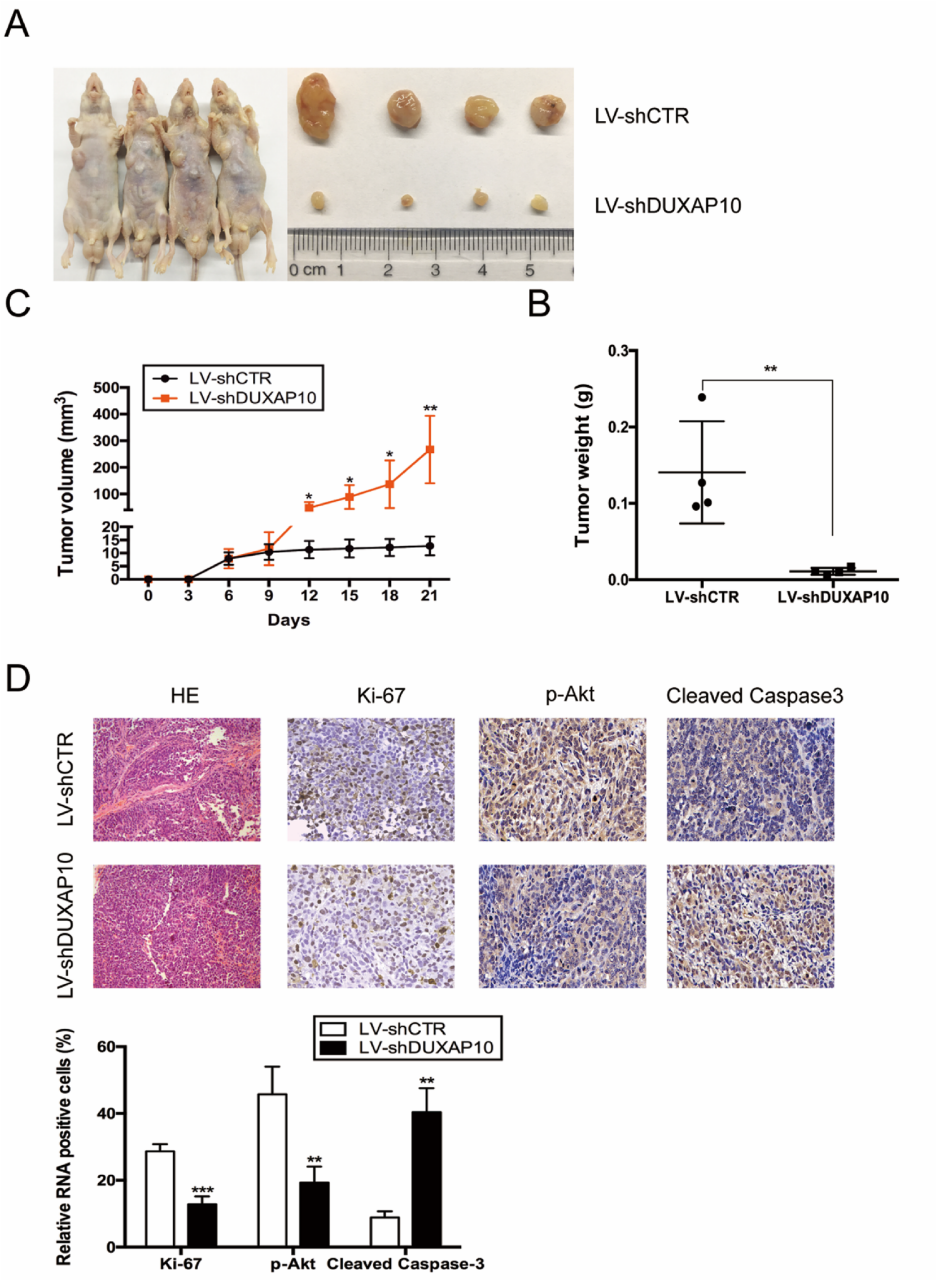
Knockdown of DUXAP10 inhibits OS cell tumorigenesis in vivo. **A** Representative gross graph of mice and xenograft tumors. **B** Tumors volume was measured by Vernier calipers. **C** Tumor weight was measured by electronic balance. **D** Representative micrographs of xenograft tumor sections stained immunohistochemically for Ki67, p-Akt, Cleaved Caspase3, and the percentage of positive tumor cells. Data are the means ± SEM of three experiments. *P < 0.05, **P < 0.01, ***P < 0.001

To further investigate the effect of DUXAP10 on the tumorigenic ability of OS cells in vivo, SAOS2 cells were transfected with slow-virus LV-sh-DUXAP10 to generate DUXAP10 stable knockdown SAOS2 cell line, and then injected into 4-week-old male immunodeficient mouse armpits subcutaneously. Three weeks later, the mice were euthanized and the xenograft tumors were removed from mouse bodies. The volume and weight of the tumors were measured. The results showed that the volume and weight of xenograft tumors from DUXAP10 stable knockdown SAOS2 cells were significantly decreased compared to the control cells (Fig. 4A–C). Immunohistochemical staining for Ki67, p-Akt, and Cleaved Caspase3 was performed and revealed that the percentage of Ki67 and p-Akt positive cells were significantly decreased in xenograft tumors from DUXAP10 stable knockdown SAOS2 cells, while the percentage of Cleaved Caspase3 positive cells showed the opposite result (Fig. 4D). These results demonstrated that knockdown of DUXAP10 can inhibit the tumorigenic ability of OS cells in vivo.

### SOX18 is identified as a critical downstream target of DUXAP10 in OS cells

**Fig. 5:**
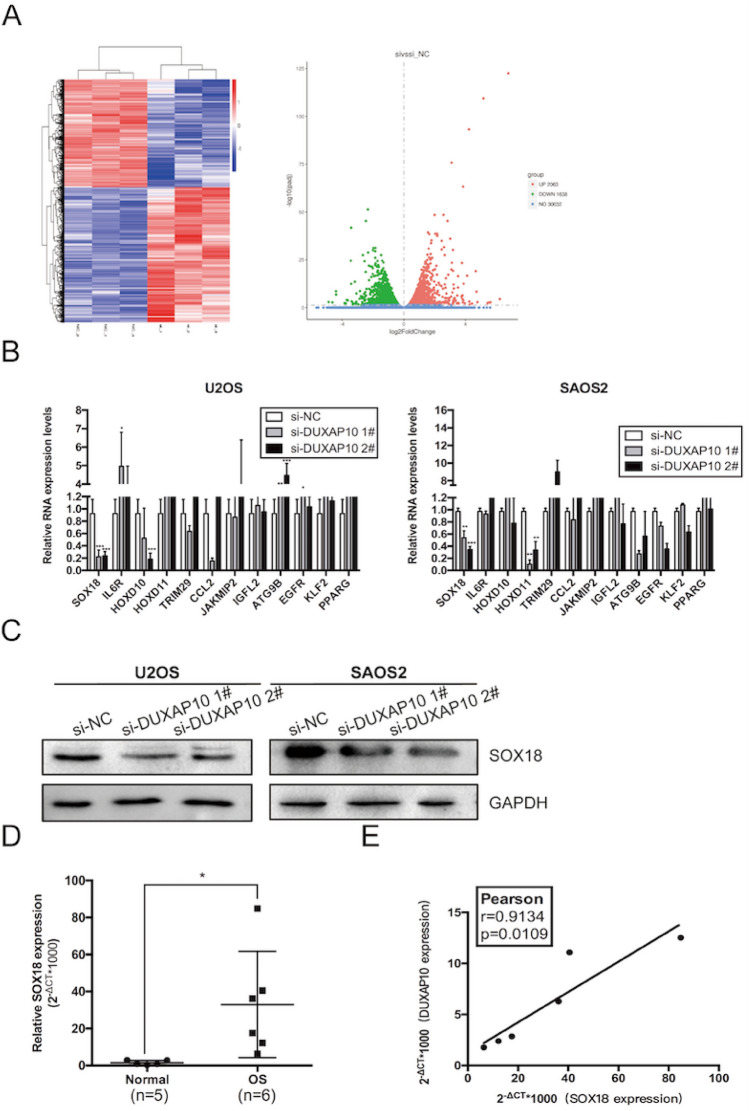
SOX18 is identified as a critical downstream target of DUXAP10 in OS cells. **A** Transcriptome high-throughput sequencing revealed differentially expressed mRNAs in DUXAP10 knockdown U2OS cells. **B** qRT-PCR assay was used to detect the changes of mRNA expression in U2OS and SAOS2 cells. **C** Western blot assay was used to detect the expression of SOX18 at protein level in U2OS and SAOS2 cells. **D** Expression of SOX18 in OS and normal bone tissues we collected. **E** Correlation analysis was used to detect the relation between DUXAP10 and SOX18 in OS tissues. Data are the means ± SEM of three experiments. *P < 0.05, **P < 0.01, ***P < 0.001

LncRNAs cannot encode proteins, but can act by regulating protein-coding genes. To accurately find the downstream target gene regulated by DUXAP10, we performed transcriptome RNA-seq in DUXAP10 knockdown and normal U2OS cells to screen differentially gene expression profile (Fig. 5A). We sorted the sequencing results by differential multiples, selected target genes related to tumor function with differential multiples > 1.5 (Table S1), and verified the sequencing results by qRT-PCR. We found the alterations of mRNA expression of SOX18 in U2OS and SAOS2 cells were consistent with the sequencing results (Fig. 5B). Western blot assay also demonstrated that the protein expression of SOX18 was down-regulated in DUXAP10 knockdown U2OS and SAOS2 cells (Fig. 5C). Next, we used qRT-PCR to detect the expression of SOX18 in the collected OS and normal bone tissues. The results showed that the expression of SOX18 mRNA was highly increased in OS tissues (Fig. 5D). Subsequently, correlation analysis revealed that there was a positive correlation between DUXAP10 and SOX18 expression in OS tissues (Fig. 5E). Meanwhile, we found that after knocking down SOX18, the proliferation ability of OS cells decreased significantly (Fig. S2). These results indicated that DUXAP10 may promote OS progression by regulating SOX18.

### DUXAP10 specifically binds to HuR in OS cells

**Fig. 6:**
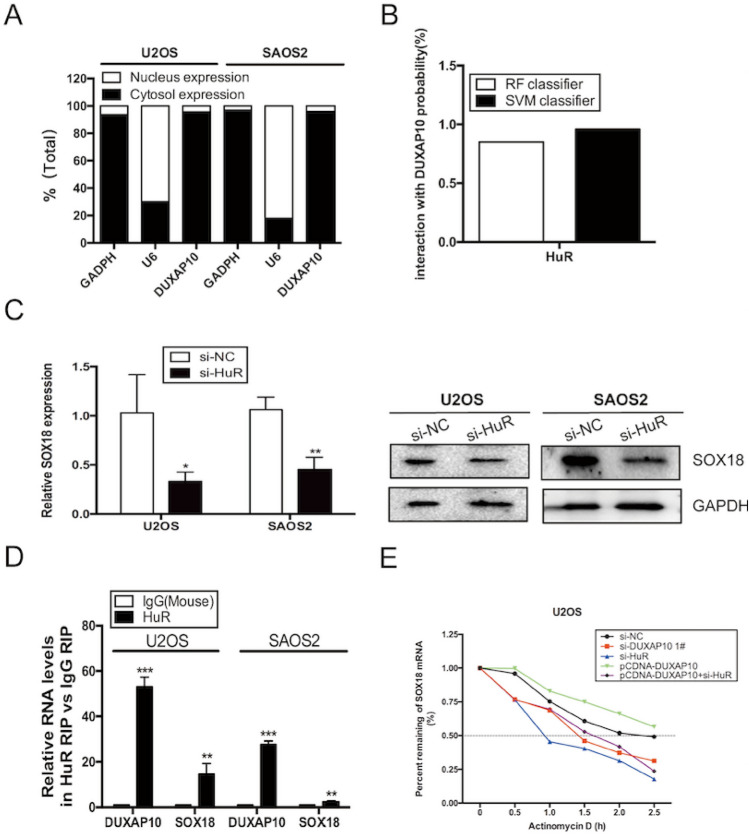
DUXAP10 specifically binds to HuR in OS cells. **A** Nucleocytoplasmic separation assay was used to detect the distribution of DUXAP10 in OS cells. B We predicted the binding ability of HuR to DUXAP10 by the online website (http://pridb.gdcb.iastate.edu/RPISeq). C qRT-PCR and Western blot assays were used to detect the expression of SOX18 in HuR knockdown U2OS and SAOS2 cells. **D** RIP assay was used to detect the degree of binding of HuR to DUXAP10 and SOX18. **E** qRT-PCR assay was used to detect the percentage of SOX18 mRNA that remained treated with actinomycin D in U2OS cells. Data are the means ± SEM of three experiments. *P < 0.05, **P < 0.01, ***P < 0.001

To explore the mechanism of DUXAP10 in regulating proliferation and migration of OS cells, we first performed nucleocytoplasmic separation assay in U2OS and SAOS2 cells, and found that DUXAP10 is mainly distributed in the cytoplasm (Fig. 6A), suggesting that DUXAP10 may function mainly at post-transcriptional levels. Recently, more and more studies have shown that lncRNAs regulate the downstream target genes by binding to proteins. To study whether DUXAP10 worked in this way, we first used the online website (http://pridb.gdcb.iastate.edu/RPISeq) to predict the collection probability of multiple RNA-binding proteins with DUXAP10. The results showed that HuR was a protein most likely to bind to DUXAP10 (Fig. S3A, Fig. 6B). Previous study had proved that HuR can not only bind to DUXAP10 but also SOX12 in glioma cells [21]. Meanwhile, we also found that the expression of SOX18 was decreased in HuR knockdown U2OS and SAOS2 cells (Fig. 6C), indicating that HuR may participate in the regulation of SOX18. Then, we performed RIP assay in OS cells, confirming that HuR bind highly to DUXAP10 and SOX18 (Fig. 6D). Previous studies have demonstrated that HuR played an important role in tumors mainly by maintaining the stability of mRNAs and slowing down their degradation [22, 23]. Therefore, we examined the effect of DUXAP10 or HuR on the stability of SOX18 mRNA. The result showed that the half-life period of SOX18 mRNA was significantly shortened in DUXAP10 or HuR knockdown U2OS cells, and extended in DUXAP10 overexpression cells. Meanwhile, we found that HuR knockdown can rescue the increased stability of SOX18 mRNA caused by DUXAP10 overexpression (Fig. 6E). In addition, after knocking down HuR, the proliferation ability of OS cells decreased significantly (Fig. S3B-D). However, knockdown of DUXAP10 did not alter HuR expression in U2OS and SAOS2 cells and vice versa (Fig. S3E&F). In addition, RIP results found that although the value of input had decreased in DUXAP10 knockdown SAOS2 cells, the decrease of the ability of HuR binding to SOX18 was more obvious (Fig. S3G). These results suggested that DUXAP10 may play a role in regulating SOX18 by recruiting and binding HuR in the cytoplasm.

### SOX18 mediates the carcinogenic process of DUXAP10 in OS

**Fig. 7:**
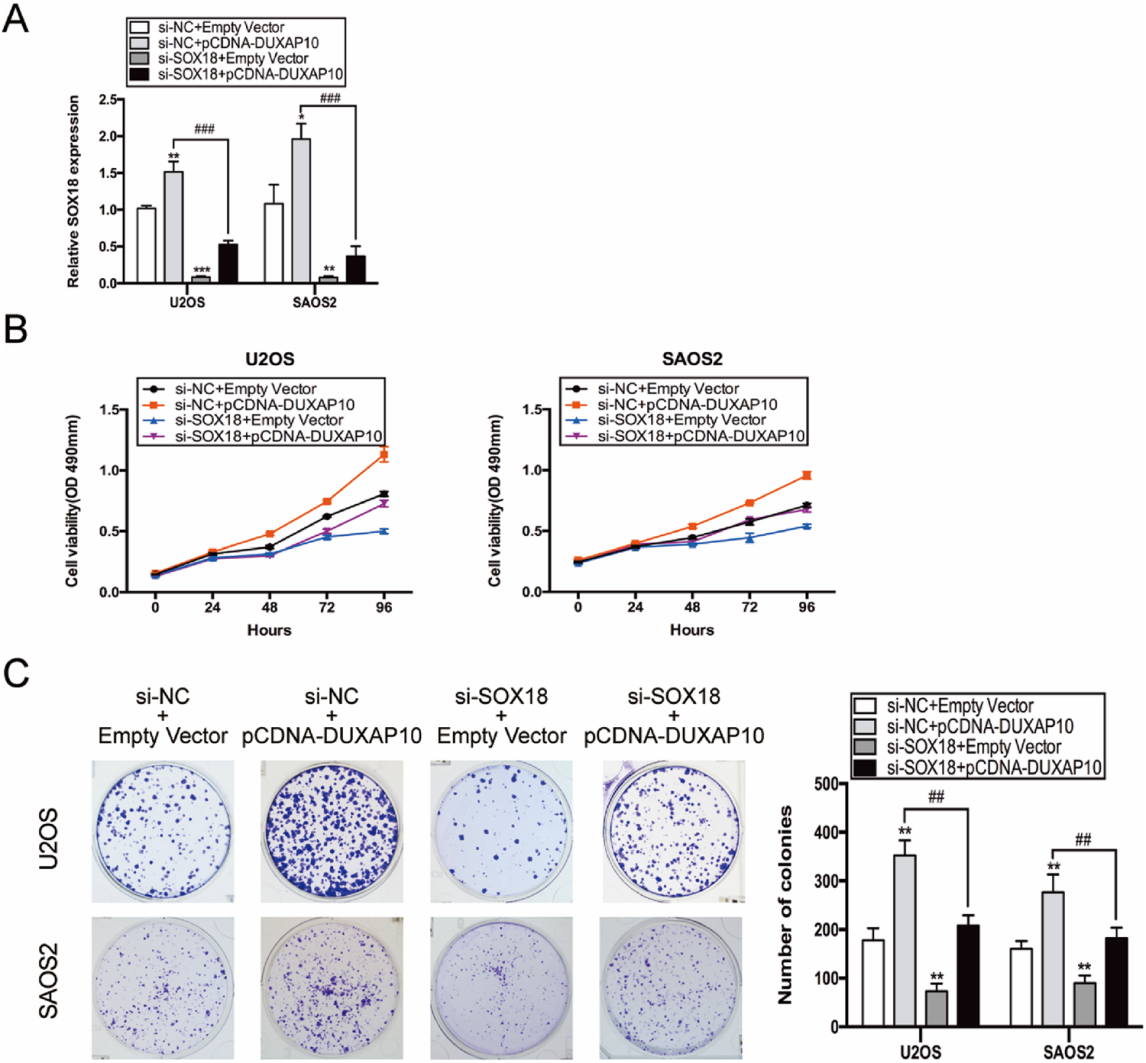
SOX18 mediated the carcinogenic process of DUXAP10 in OS cells. **A** Relative SOX18 expression in co-transfection experiments. **B**, **C** MTT and colony formation assays were used to detect the effect of SOX18 knockdown on the proliferation activity of U2OS and SAOS2 cells with DUXAP10 overexpression. Data are the means ± SEM of three experiments. *P < 0.05, **P < 0.01, ***P < 0.001

To further determine whether SOX18 mediated the carcinogenic process of DUXAP10, U2OS and SAOS2 cells were transfected with either pCDNA-DUXAP10 or both pCDNA-DUXAP10 and si-SOX18. qRT-PCR results showed that overexpression of DUXAP10 resulted in increased expression of SOX18, but after co-transfection of si-SOX18, the expression of SOX18 decreased significantly (Fig. 7A). MTT and colony formation assays were performed to detect their effects on OS cell proliferation. The result showed that knockdown of SOX18 largely reversed increased proliferation of OS cells induced by DUXAP10 overexpression (Fig. 7B, C). Previous studies have reported that SOX18 played an important role in some tumors by regulating cell cycle. To explore whether SOX18 regulates the proliferation of OS cells by affecting cell cycle, we examined the cell cycle changes by flow cytometry and cell cycle regulators by qRT-PCR in SOX18 knockdown U2OS and SAOS2 cells. The results showed that SOX18 has a significant effect on OS cell cycle (Fig. S4). These results demonstrated that SOX18 mediated the carcinogenic process of DUXAP10 in OS cells by regulating OS cell cycle.

## Discussion

In recent years, with the rapid development of sequencing technology, long non-coding RNAs have been found to participate in various biological processes by regulating the expression of protein-coding genes [24]. Disorders of lncRNAs lead to the development of a variety of diseases, especially tumors [25–29]. In this study, lncRNA DUXAP10 was first discovered to be highly expressed in OS and affect proliferation and migration of OS cells. Previous studies have demonstrated that DUXAP10 plays a carcinogenic role in non-small cell lung cancer [15], colorectal cancer [16], and gastric cancer [17] by binding LSD1; DUXAP10 promotes tumor progression in pancreatic cancer [18] and esophageal squamous cell carcinoma [19] by binding EZH2; DUXAP10 promotes cell proliferation by inhibiting PI3K/Akt/mTOR signaling pathway in bladder cancer [20]. In this study, we analyzed the expression of DUXAP10 in human OS tissues using raw microarray data downloaded from GEO (GSE28424). We found that DUXAP10 expressed highly in OS tissues compared to normal human bone tissue. More importantly, to date, no studies have revealed the molecular mechanisms and downstream targets of DUXAP10 in OS. Here, we demonstrate that knockdown of DUXAP10 inhibits OS cell proliferation, metastasis, and promotes apoptosis. Animal experiments showed that knockdown of DUXAP10 inhibited tumorigenesis of OS cells. RNA-seq results showed that DUXAP10 regulates the expression of proliferation and apoptosis-related genes after knocking down DUXAP10 in OS cells. In addition, this study provides the first evidence that DUXAP10, which is highly enriched in the cytoplasm, interacts with HuR and inhibits the degradation of SOX18 mRNA, thereby up-regulating the expression of SOX18 and promoting OS proliferation and metastasis. This study revealed that DUXAP10 plays a carcinogenic role in human OS cells, which helped to improve the molecular mechanism of OS development and provided new ideas for the clinical diagnosis and treatment of OS.

We first analyzed the OS case–control chip in the GEO database and found that lncRNA DUXAP10 was significantly up-regulated in OS, and then, we verified this alteration in OS tissues and cells. Functional experiments demonstrated that DUXAP10 could promote the proliferation, migration, and invasion of OS cells, regulate cell cycle, and inhibit cell apoptosis in vitro and enhance the tumorigenic ability of OS cells in vivo. These results indicate that DUXAP10 is an important lncRNA in the progression of OS.

DUXAP10, as an lncRNA, does not encode protein, but acts by regulating the protein-coding genes. To find the target gene regulated by DUXAP10 exactly, we performed high-throughput sequencing of transcriptome in DUXAP10 knockdown U2OS cells, and analyzed the sequencing results. It was found that the expression of SOX18 was significantly decreased in DUXAP10 knockdown U2OS and SAOS2 cells at both mRNA and protein levels, indicating that DUXAP10 may promote OS progression by regulating the expression of SOX18.

It is well known that lncRNAs play different roles depending on their subcellular localization. Different subcellular localization of LncRNA often indicates its different regulatory effects. In recent years, it has become a hotspot that lncRNAs specifically bind to protein to play a regulatory role in tumors [30–33]. To study the molecular mechanism of DUXAP10 in OS, we found that DUXAP10 was mainly distributed in the cytoplasm by nucleocytoplasmic separation assay, suggesting that DUXAP10 mainly function at the post-transcriptional level in OS. Furthermore, we demonstrated that DUXAP10 was able to specifically bind to HuR in OS cells by RIP assay. HuR, also known as ELAVL1, is an RNA-binding protein. It has been reported in previous studies to be highly expressed in OS [34] and to maintain mRNAs stability at the post-transcriptional level to promote their translation, thereby promoting tumor progression [35]. In addition, HuR can be an oncogene independently to mediate tumor progression by regulating nuclear import of proteins [36–41]. Our results demonstrated that HuR also bound to SOX18 and the expression of SOX18 was decreased in HuR knockdown OS cells. However, though DUXAP10 recruited and bound HuR, it did not affect HuR expression in OS cells, suggesting that DUXAP10 may participate in OS progression by binding HuR to co-regulate the transcription of SOX18. Then, we surprisingly found that the stability of SOX18 mRNA was significantly decreased in DUXAP10 or HuR knockdown cells, indicating that the stability of SOX18 mRNA is regulated by “DUXAP10-HuR” complex.

Sex-determining region Y-box 18 (SOX18) is a member of the sex-determining region of the Y-chromosome-related high-mobility group box (SOX) family of transcription factor. There are some studies have reported that SOX18 functioned as an oncogene in tumors by regulating cell cycle and apoptosis [42–44]. However, its molecule mechanism in osteosarcoma has not yet been discovered [45, 46]. In this study, our results revealed that SOX18 has a significant effect on the cell cycle of OS cells. Furthermore, we demonstrated that SOX18 knockdown largely reversed increased proliferation of OS cells induced by DUXAP10 overexpression. The results from our study indicate that SOX18 mediates the DUXAP10 carcinogenesis process by regulating the cell cycle.

**Fig. 8:**
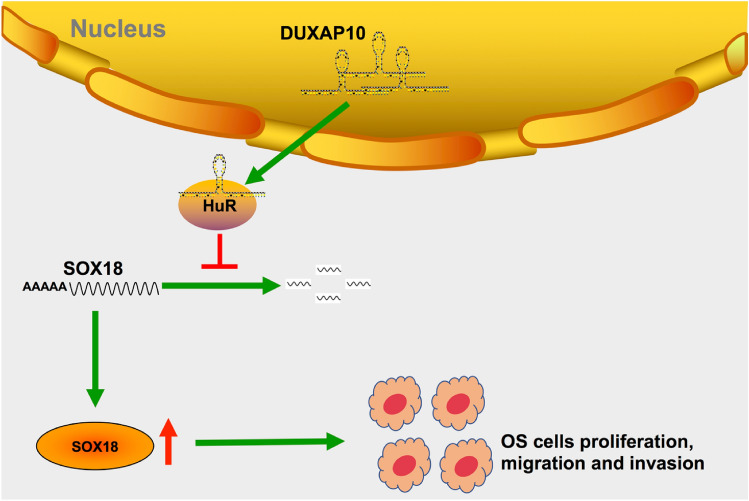
Schematic diagram of DUXAP10 promoting the proliferation, migration, and invasion of osteosarcoma cells

In conclusion, in this study, we found that lncRNA DUXAP10 is highly expressed in OS, and can promote proliferation, migration, and invasion of OS cells and inhibit their apoptosis. Mechanically, lncRNA DUXAP10 recruits HuR to maintain the stability of SOX18 mRNA, promote SOX18 translation, and then affect OS cell cycle, and finally promote the progression of OS (Fig. 8). However, due to the lack of clinical OS tissues and pathological data, the relationship between DUXAP10 expression and OS prognosis has not been confirmed. Therefore, more efforts are needed to apply lncRNAs to the diagnosis and treatment of osteosarcoma.

## Supplementary Information

Below is the link to the electronic supplementary material.

Supplementary file1 (DOCX 2932 KB)
