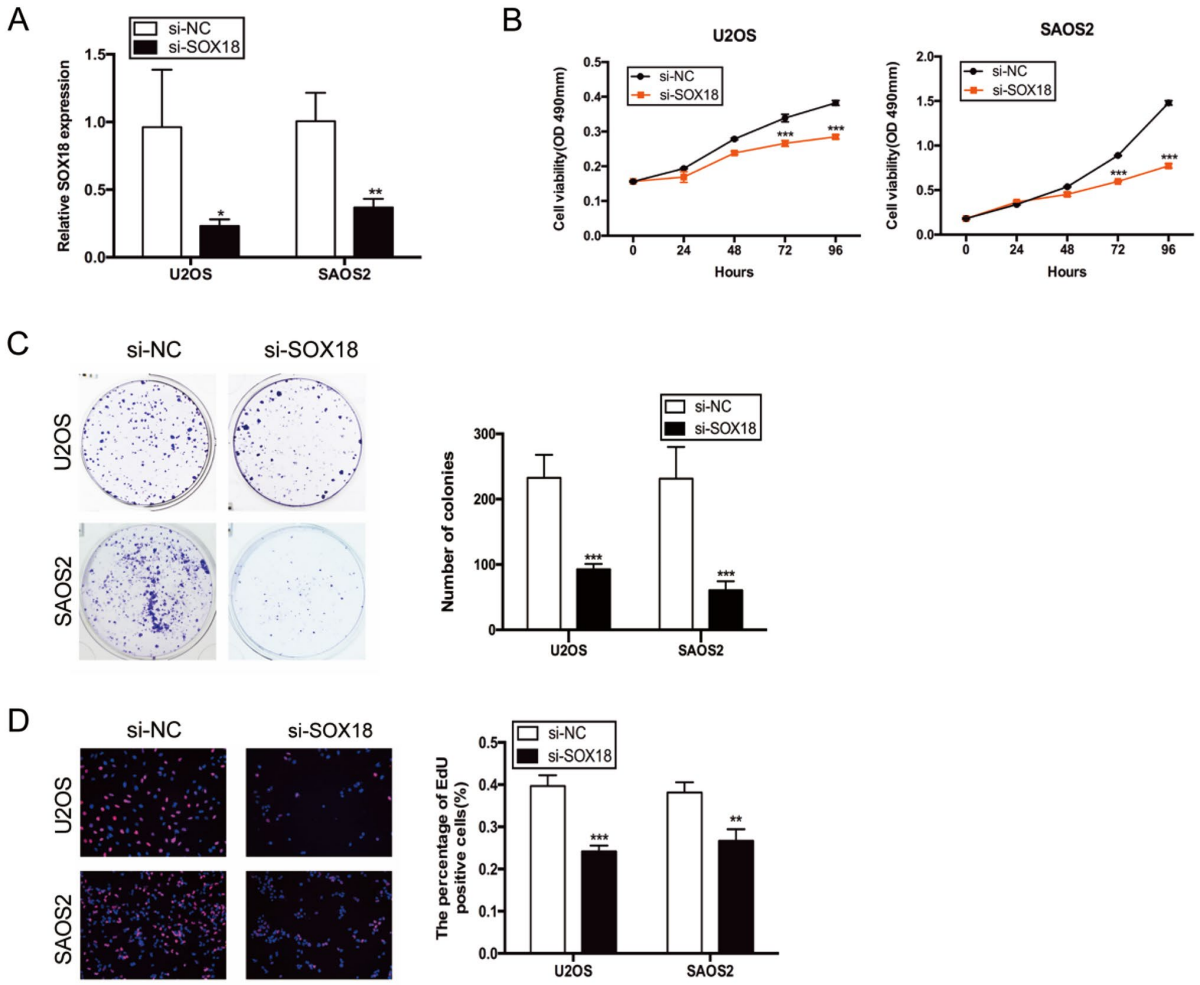# Correction: Long non-coding RNA DUXAP10 exerts oncogenic properties in osteosarcoma by recruiting HuR to enhance SOX18 mRNA stability

**DOI:** 10.1007/s13577-025-01281-0

**Published:** 2025-09-12

**Authors:** Guantong Wang, Qian Zhang, Qinjue Wang, Jing Wang, Lulu Chen, Qiang Sun, Dengshun Miao

**Affiliations:** 1 Department of Orthopedics, Nanjing First Hospital, Nanjing Medical University, Nanjing, China; 2 State Key Laboratory of Reproductive Medicine, Research Center for Bone and Stem Cells, Key Laboratory for Aging and Disease, Nanjing Medical University, Nanjing, China

**Correction: Human Cell (2022) 35:1939–1951** 10.1007/s13577-022-00772-8

In Fig. 3B of this article, the image for the pCDNA-DUXAP10 group in SAOS2 cells was mistakenly labeled as si-NC. It should have appeared as in this correction. Additionally, in Figure S2C of this article, the SAOS2-cell image in Fig. S2C was inadvertently duplicated from the SAOS2 panel in Fig. 2C. It should also have appeared as in this correction.

**Incorrect version:**

(1) Figure 3
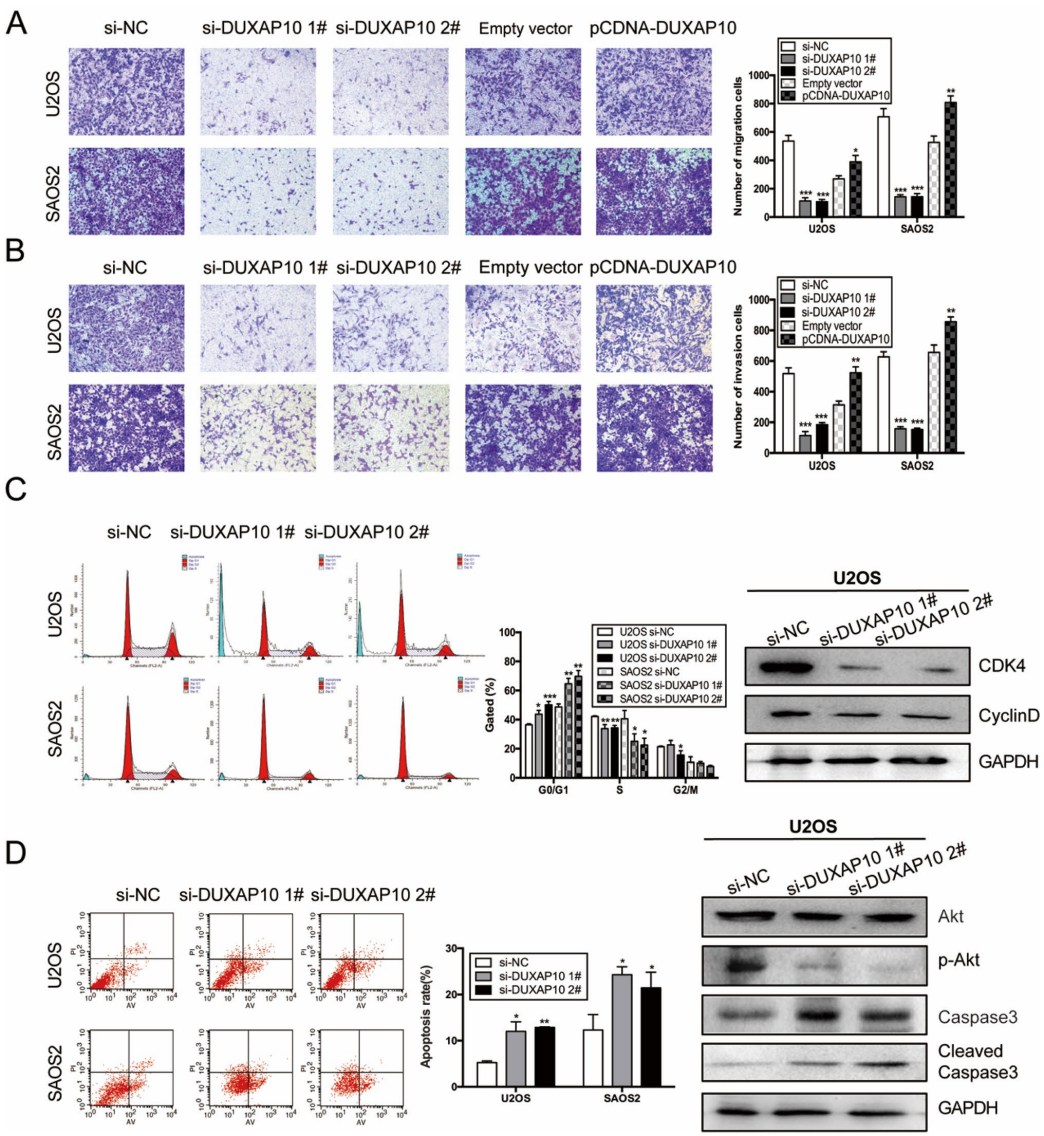


(2) Figure S2
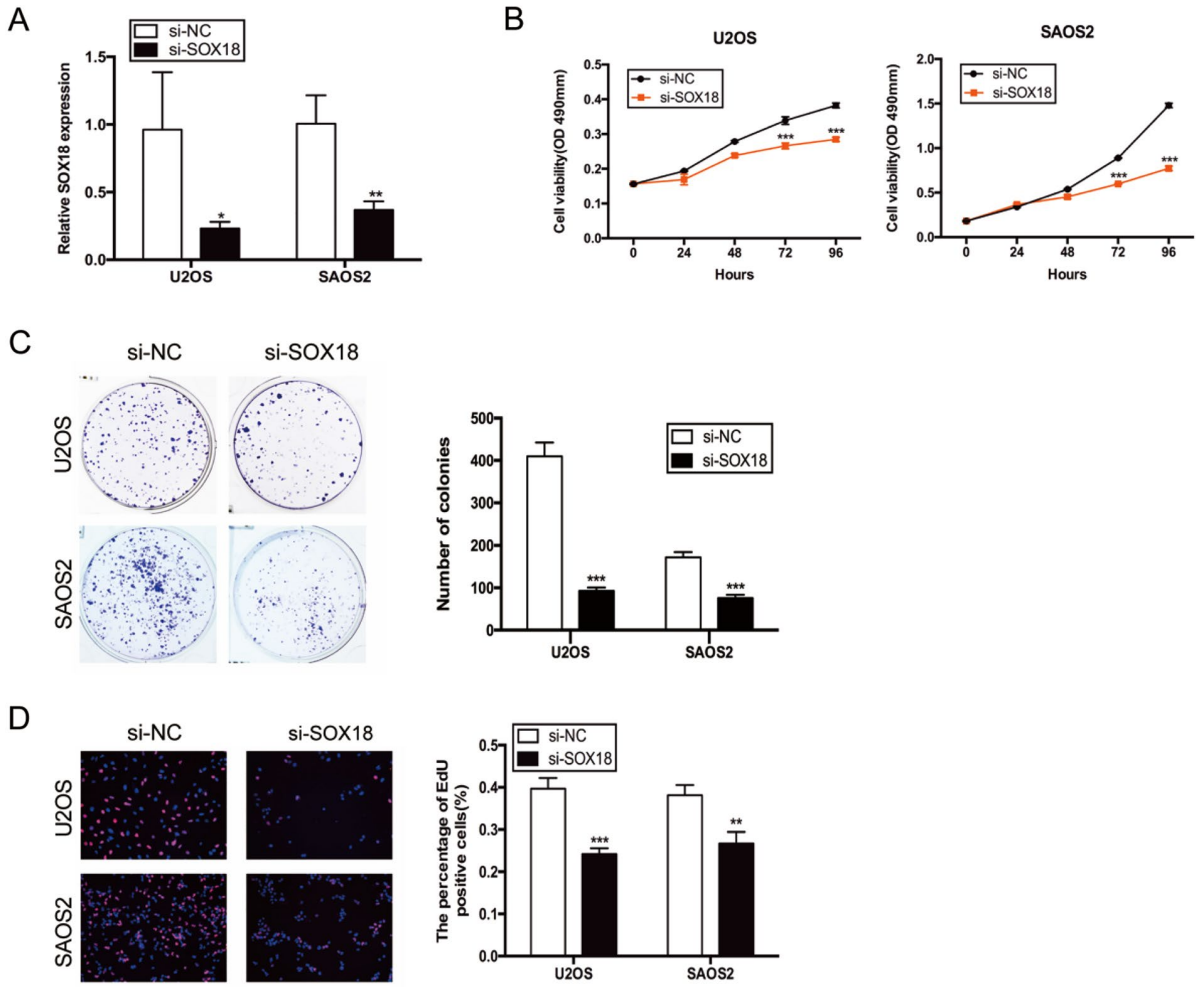


**Correct version:**

(1) Figure 3
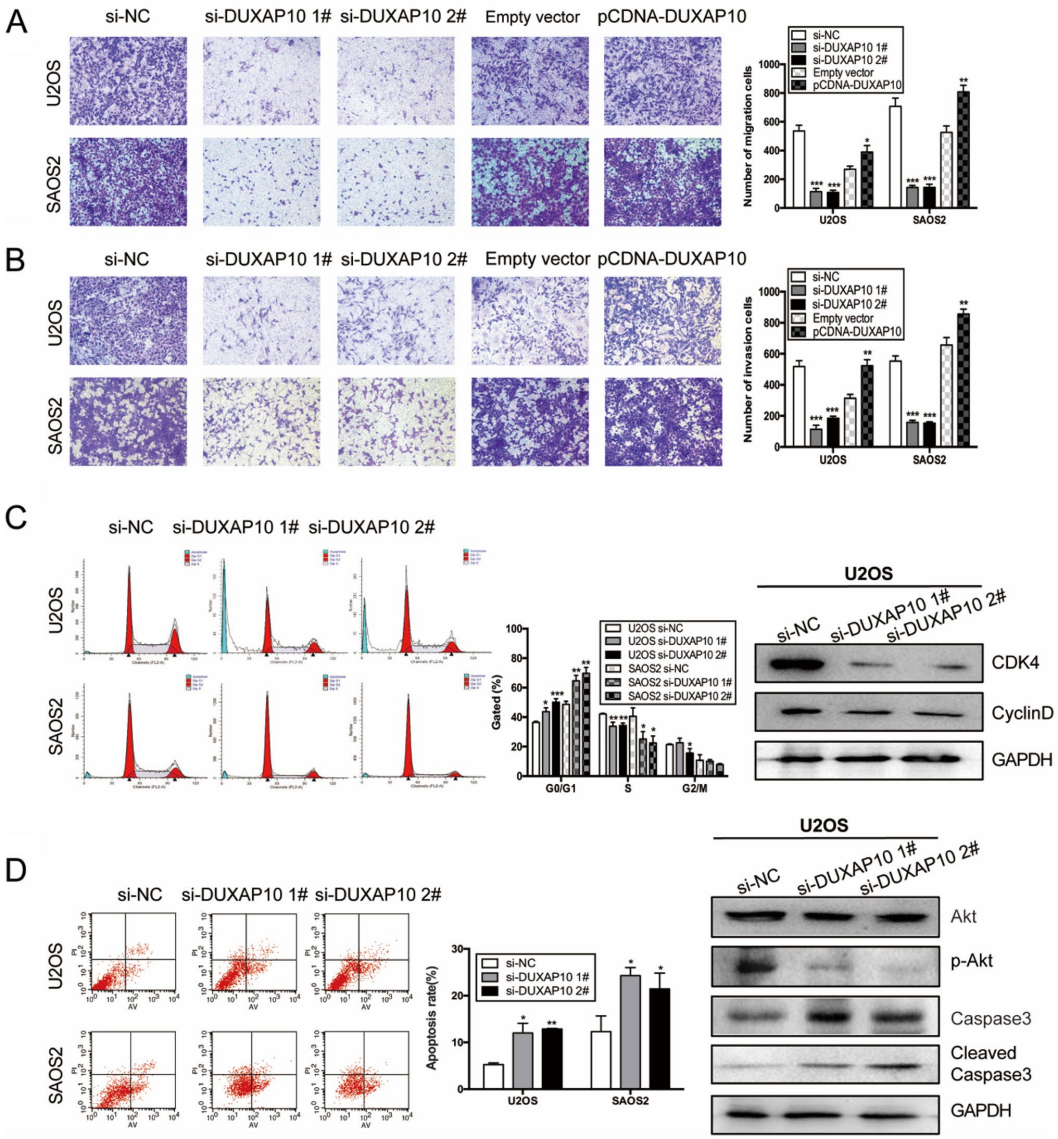


(2) Figure S2