# Supplementary material for: Long non-coding RNA DUXAP10 exerts oncogenic properties in osteosarcoma by recruiting HuR to enhance SOX18 mRNA stability
Source: Hum Cell. 2022 Sep 2;35(6):1939–51. doi: 10.1007/s13577-022-00772-8 (PMC9515053; doi:10.1007/s13577-022-00772-8)
Supplement: Supplementary file 1 — Supplementary file1 (DOCX 2932 KB) [file 13577_2022_772_MOESM1_ESM.docx]

**Long noncoding RNA DUXAP10 exerts oncogenic properties in osteosarcoma by recruiting HuR to enhance SOX18 mRNA stability**

Guantong Wang^1^, Qian Zhang ^2^, Qinjue Wang ^1^, Jing Wang^2^, Lulu Chen^2^, Qiang Sun^1^ and Dengshun Miao^2^

^1^Department of Orthopedics, Nanjing First Hospital, Nanjing Medical University, Nanjing, China; ^2^State Key Laboratory of Reproductive Medicine, Research Center for Bone and Stem Cells, Key Laboratory for Aging & Disease; Nanjing Medical University, Nanjing, China.

**Corresponding Authors:**

Dr. Qiang Sun

Department of Orthopedics,

Nanjing First Hospital, Nanjing Medical University,

Nanjing, China

E-mail: sunqiangsubmission@163.com

Tel & FAX: 011-86-25-8545-3104

ORCID: sunqiangsubmission@163.com

Or Dr. Dengshun Miao

State Key Laboratory of Reproductive Medicine,

Research Center for Bone and Stem Cells,

Key Laboratory for Aging & Disease,

Nanjing Medical University,

Nanjing, China.

E-mail: [dsmiao@njmu.edu.cn](mailto:dsmiao@njmu.edu.cn)

Tel & FAX: 011-86-25-8686-9377

| **Table S1. Primers used in this study** | | |
| --- | --- | --- |
| **Name** | **Forward-primer** | **Reverse-primer** |
| GAPDH | GGAGCGAGATCCCTCCAAAAT | GGCTGTTGTCATACTTCTCATGG |
| DUXAP10 | GAGAAGCAGTGGTGGGTTCC | GAGCAACACAGATGAACCGC |
| U6 | CTCGCTTCGGCAGCACA | AACGCTTCACGAATTTGCGT |
| HuR | GGGTGACATCGGGAGAACG | CTGAACAGGCTTCGTAACTCAT |
| SOX18 | CGCGTGTATGTTTGGTTC | ATGTAACCCTGGCAACTC |
| IL6R | CTCCTGCCAGTTAGCAGTCC | TCTTGCCAGGTGACACTGAG |
| HOXD10 | GACATGGGGACCTATGGAATGC | CGGATCTGTCCAACTGTCTACT |
| HOXD11 | TCGACCAGTTCTACGAGGCA | AAAAACTCGCGTTCCAGTTCG |
| TRIM29 | CTGTTCGCGGGCAATGAGT | TGCCTTCCATAGAGTCCATGC |
| CCL2 | CAGCCAGATGCAATCAATGCC | TGGAATCCTGAACCCACTTCT |
| JAKMIP2 | TCCAAGAAAGGGCGAAATAAGG | TGCAGCTCTATCTGAATGTCTGT |
| IGFL2 | GTGGCATCGTTGAGGAGTG | CACGTCCCTCTCGGACTTG |
| ATG9B | CCCCTCATACAAGAAGCTCCC | TGCAGGTTGAGCCTGTGTTG |
| EGFR | AGGCACGAGTAACAAGCTCAC | ATGAGGACATAACCAGCCACC |
| KLF2 | TTCGGTCTCTTCGACGACG | TGCGAACTCTTGGTGTAGGTC |
| PPARG | GGGATCAGCTCCGTGGATCT | TGCACTTTGGTACTCTTGAAGTT |
| P16 | GGGTTTTCGTGGTTCACATCC | CTAGACGCTGGCTCCTCAGTA |
| P27 | AACGTGCGAGTGTCTAACGG | CCCTCTAGGGGTTTGTGATTCT |
| CDK4 | ATGGCTACCTCTCGATATGAGC | CATTGGGGACTCTCACACTCT |
| CDK6 | GCTGACCAGCAGTACGAATG | GCACACATCAAACAACCTGACC |

| **Table S2. si-RNAs used in this study** | |
| --- | --- |
| **Name** | **Sense (5’-3’)** |
| si-NC | UUCUCCGAACGUGUCACGUTT |
| si-DUXAP10 1# | UGGCUGGAGAAUAUUUCCUAGUGUA |
| si-DUXAP10 2# | CCAAAGGUUUCUGGCUGAAACAUAU |
| si-DUXAP10 3# | CAGCAUACUUCAAAUUCACAGCAAA |
| si-HuR | CACGCUGAACGGCUUGAGGTT |
| si-SOX18 | TACCACGTGGCACTGGCCATT |

| **Table S3. Differential abundance of mRNAs (Log2FoldChange >1.5) in si-DUXAP10 U2OS cells** | | | | |
| --- | --- | --- | --- | --- |
| **Gene_name** | **Log2FoldChange** | **P value** | **Gene_biotype** | **Style** |
| NOS3 | 5.16127827 | 4.48E-114 | protein_coding | up |
| AGXT | 4.71749662 | 9.68E-11 | protein_coding | up |
| ASB18 | 4.41428631 | 0.00088601 | protein_coding | up |
| NAA11 | 4.26769211 | 0.00403904 | protein_coding | up |
| CD163 | 4.24417733 | 5.36E-05 | protein_coding | up |
| RNASE7 | 4.10777422 | 1.35E-19 | protein_coding | up |
| HMOX1 | 3.84631838 | 1.31E-67 | protein_coding | up |
| IGFN1 | 3.81048249 | 1.15E-08 | protein_coding | up |
| IL13RA2 | 3.77265284 | 1.23E-26 | protein_coding | up |
| PSG6 | 3.47924416 | 0.00034204 | protein_coding | up |
| TNFSF8 | 3.46898896 | 1.34E-08 | protein_coding | up |
| FAM180A | 3.44848705 | 0.00075522 | protein_coding | up |
| PNLIPRP3 | 3.30357432 | 4.73E-07 | protein_coding | up |
| PRR9 | 3.2747909 | 1.97E-06 | protein_coding | up |
| TMEM233 | 3.25567805 | 1.89E-06 | protein_coding | up |
| KCNU1 | 3.2347106 | 0.00015536 | protein_coding | up |
| ARL14 | 3.23312346 | 8.67E-15 | protein_coding | up |
| ATG9B | 3.20616705 | 3.70E-05 | protein_coding | up |
| PDCD1LG2 | 3.18944986 | 1.06E-33 | protein_coding | up |
| SERF1B | 3.18444173 | 0.00034734 | protein_coding | up |
| DHRS9 | 3.15441441 | 2.63E-10 | protein_coding | up |
| CD22 | 3.10123419 | 3.17E-80 | protein_coding | up |
| DCDC2C | 3.08573067 | 4.41E-23 | protein_coding | up |
| ALOXE3 | 3.0069064 | 8.70E-40 | protein_coding | up |
| DIRC3 | 2.96309709 | 2.78E-07 | protein_coding | up |
| PDE7B | 2.92372857 | 1.59E-06 | protein_coding | up |
| C1QL2 | 2.73211976 | 3.16E-18 | protein_coding | up |
| SLC28A3 | 2.73138551 | 4.10E-05 | protein_coding | up |
| CPXM2 | 2.679922 | 5.49E-07 | protein_coding | up |
| KLF15 | 2.66144752 | 7.12E-15 | protein_coding | up |
| MMP3 | 2.58010506 | 1.34E-52 | protein_coding | up |
| KLHL38 | 2.54029341 | 0.00011242 | protein_coding | up |
| SP140 | 2.46110114 | 1.26E-38 | protein_coding | up |
| ZNF554 | 2.45894724 | 5.70E-34 | protein_coding | up |
| SIGLEC9 | 2.45493242 | 0.00021923 | protein_coding | up |
| MYCT1 | 2.44641129 | 7.16E-14 | protein_coding | up |
| THSD7A | 2.32506422 | 6.89E-05 | protein_coding | up |
| ABCC9 | 2.28977249 | 8.18E-09 | protein_coding | up |
| ESM1 | 2.25834077 | 8.97E-16 | protein_coding | up |
| BEST3 | 2.220404 | 0.00010534 | protein_coding | up |
| CD177 | 2.19082306 | 3.48E-11 | protein_coding | up |
| DRC7 | 2.18798911 | 0.00814699 | protein_coding | up |
| GULP1 | 2.16830822 | 2.61E-41 | protein_coding | up |
| IL6R | 2.16254162 | 1.96E-42 | protein_coding | up |
| UNC5A | 2.14777691 | 3.65E-20 | protein_coding | up |
| PSG4 | 2.11575505 | 1.60E-14 | protein_coding | up |
| DGKI | 2.11052835 | 4.45E-25 | protein_coding | up |
| IL1RAPL1 | 2.10697676 | 6.99E-10 | protein_coding | up |
| XKR3 | 2.09712938 | 0.00137047 | protein_coding | up |
| NT5DC4 | 2.06031171 | 0.00068338 | protein_coding | up |
| LCP1 | 2.04805328 | 1.42E-42 | protein_coding | up |
| BATF2 | 2.02457678 | 3.28E-07 | protein_coding | up |
| ACSM4 | 2.01022748 | 0.00228398 | protein_coding | up |
| VAV1 | 2.00330766 | 4.13E-10 | protein_coding | up |
| EVI2A | 2.00203269 | 1.68E-05 | protein_coding | up |
| LAMB3 | 1.99600739 | 1.60E-52 | protein_coding | up |
| COL5A3 | 1.9895261 | 0.00318151 | protein_coding | up |
| PATL2 | 1.98913102 | 0.00063108 | protein_coding | up |
| MPZ | 1.98857461 | 0.00316575 | protein_coding | up |
| ACSL5 | 1.9846596 | 0.00016266 | protein_coding | up |
| C4orf19 | 1.97556783 | 1.82E-16 | protein_coding | up |
| SIGLEC15 | 1.96503919 | 1.18E-11 | protein_coding | up |
| IL15RA | 1.95324493 | 5.31E-13 | protein_coding | up |
| FAM196B | 1.92610925 | 1.03E-07 | protein_coding | up |
| PTPRR | 1.92553363 | 1.10E-11 | protein_coding | up |
| CBLN4 | 1.91277468 | 0.00095296 | protein_coding | up |
| CD163L1 | 1.89690912 | 1.18E-22 | protein_coding | up |
| CR2 | 1.89155455 | 0.0002411 | protein_coding | up |
| LY96 | 1.86420422 | 7.94E-12 | protein_coding | up |
| TRPV2 | 1.85005366 | 2.55E-08 | protein_coding | up |
| ANTXR2 | 1.84760125 | 1.75E-29 | protein_coding | up |
| HTN1 | 1.83792612 | 1.71E-06 | protein_coding | up |
| DPP4 | 1.81756783 | 8.20E-21 | protein_coding | up |
| DRD2 | 1.81540036 | 2.55E-06 | protein_coding | up |
| TM4SF19 | 1.81415989 | 4.06E-11 | protein_coding | up |
| BACH2 | 1.7962469 | 2.52E-17 | protein_coding | up |
| ITGA2 | 1.79451343 | 9.97E-19 | protein_coding | up |
| DUSP5 | 1.79356219 | 2.28E-29 | protein_coding | up |
| ADAMTS9 | 1.78766222 | 6.34E-07 | protein_coding | up |
| TMEM132D | 1.78482374 | 4.32E-07 | protein_coding | up |
| FOSL1 | 1.77360639 | 1.99E-37 | protein_coding | up |
| REPS2 | 1.7594318 | 9.08E-11 | protein_coding | up |
| NRIP3 | 1.74467719 | 1.45E-31 | protein_coding | up |
| ARHGAP27 | 1.74199009 | 1.78E-18 | protein_coding | up |
| KLHL21 | 1.73441041 | 2.30E-26 | protein_coding | up |
| GEM | 1.73200564 | 1.57E-28 | protein_coding | up |
| ADCY8 | 1.73170015 | 4.89E-07 | protein_coding | up |
| C3AR1 | 1.71872493 | 7.96E-05 | protein_coding | up |
| BASP1 | 1.71355112 | 6.92E-06 | protein_coding | up |
| RNF103-CHMP3 | 1.70512632 | 8.89E-12 | protein_coding | up |
| EPHB1 | 1.70149633 | 4.43E-15 | protein_coding | up |
| HRK | 1.68568723 | 1.83E-21 | protein_coding | up |
| KLF2 | 1.67886774 | 2.58E-12 | protein_coding | up |
| CNTN1 | 1.67810445 | 1.36E-11 | protein_coding | up |
| RENBP | 1.67078823 | 4.15E-15 | protein_coding | up |
| GOLGA7B | 1.66579948 | 0.00166393 | protein_coding | up |
| FGF5 | 1.65667229 | 1.08E-12 | protein_coding | up |
| ENPP5 | 1.65576859 | 7.19E-10 | protein_coding | up |
| CCL3 | 1.64997065 | 0.00019499 | protein_coding | up |
| HMGA2 | 1.64988095 | 2.62E-42 | protein_coding | up |
| SLAMF7 | 1.6479948 | 1.13E-12 | protein_coding | up |
| RRAGD | 1.64797338 | 6.18E-22 | protein_coding | up |
| PSG5 | 1.64547573 | 0.00024718 | protein_coding | up |
| PRSS8 | 1.64451618 | 2.90E-05 | protein_coding | up |
| PLEKHM1 | 1.64410226 | 1.67E-29 | protein_coding | up |
| NFATC2 | 1.64058867 | 1.22E-14 | protein_coding | up |
| TRIB1 | 1.63766851 | 9.40E-30 | protein_coding | up |
| TAF4B | 1.63539218 | 1.64E-06 | protein_coding | up |
| HCN1 | 1.62810205 | 8.84E-09 | protein_coding | up |
| ADAM12 | 1.62444162 | 1.60E-24 | protein_coding | up |
| G0S2 | 1.61776706 | 3.55E-08 | protein_coding | up |
| CCRL2 | 1.61719889 | 1.50E-06 | protein_coding | up |
| COL19A1 | 1.61588807 | 0.00366609 | protein_coding | up |
| PPP1R15A | 1.610036 | 1.82E-34 | protein_coding | up |
| TPRG1L | 1.59866769 | 1.02E-25 | protein_coding | up |
| C14orf105 | 1.59678505 | 0.00647134 | protein_coding | up |
| ZNF469 | 1.59266913 | 9.10E-16 | protein_coding | up |
| ANKRD13B | 1.57993414 | 6.74E-24 | protein_coding | up |
| TGFBR2 | 1.56153474 | 2.88E-43 | protein_coding | up |
| LONRF1 | 1.55573518 | 4.12E-15 | protein_coding | up |
| PAX8 | 1.54198977 | 2.21E-05 | protein_coding | up |
| FZD8 | 1.53813648 | 2.31E-13 | protein_coding | up |
| PPARG | 1.53391274 | 9.87E-26 | protein_coding | up |
| EGFR | 1.53167994 | 1.20E-19 | protein_coding | up |
| SH2D5 | 1.52872357 | 2.36E-13 | protein_coding | up |
| AXL | 1.5257373 | 3.19E-31 | protein_coding | up |
| KCNH2 | 1.5247499 | 1.19E-16 | protein_coding | up |
| MAP7 | 1.51940832 | 3.69E-10 | protein_coding | up |
| HTN3 | 1.51843831 | 0.00246712 | protein_coding | up |
| C1orf61 | 1.51416975 | 0.00068199 | protein_coding | up |
| EPG5 | 1.51413842 | 1.66E-08 | protein_coding | up |
| UAP1L1 | 1.51187717 | 2.79E-28 | protein_coding | up |
| TTC9B | 1.51145033 | 1.68E-05 | protein_coding | up |
| NDRG4 | 1.51088195 | 3.75E-24 | protein_coding | up |
| IRX6 | 1.5075999 | 2.49E-06 | protein_coding | up |
| SMIM29 | 1.50224512 | 1.09E-22 | protein_coding | up |
| LMTK3 | 1.50076529 | 7.24E-09 | protein_coding | up |
| SYNM | -1.5000151 | 0.00044633 | protein_coding | down |
| PAFAH1B2 | -1.5022025 | 4.83E-21 | protein_coding | down |
| SYT8 | -1.5023522 | 0.00358737 | protein_coding | down |
| RADIL | -1.5032071 | 0.00462368 | protein_coding | down |
| PDS5B | -1.503248 | 0.00523102 | protein_coding | down |
| DDX21 | -1.5070049 | 8.78E-10 | protein_coding | down |
| GPR55 | -1.5145523 | 4.29E-05 | protein_coding | down |
| LIFR | -1.517075 | 7.61E-10 | protein_coding | down |
| HMGCS1 | -1.5222873 | 2.93E-14 | protein_coding | down |
| BPIFB4 | -1.5296888 | 2.45E-11 | protein_coding | down |
| SORBS2 | -1.5312565 | 2.06E-05 | protein_coding | down |
| SLITRK2 | -1.5326918 | 1.01E-05 | protein_coding | down |
| CDHR1 | -1.5394576 | 4.14E-07 | protein_coding | down |
| RASGRP4 | -1.5410237 | 0.00072923 | protein_coding | down |
| FZD9 | -1.5504612 | 0.00138416 | protein_coding | down |
| GGT5 | -1.5571304 | 1.23E-13 | protein_coding | down |
| ATOH8 | -1.5646588 | 7.62E-13 | protein_coding | down |
| TLL1 | -1.5667242 | 1.25E-06 | protein_coding | down |
| TEX14 | -1.5677156 | 0.00867226 | protein_coding | down |
| RCOR2 | -1.5692156 | 1.64E-08 | protein_coding | down |
| ZNF708 | -1.570432 | 0.00023557 | protein_coding | down |
| MMGT1 | -1.5711198 | 1.76E-20 | protein_coding | down |
| SLC26A9 | -1.5745516 | 0.00218742 | protein_coding | down |
| GPRIN3 | -1.5749553 | 0.00304038 | protein_coding | down |
| FRRS1L | -1.5750525 | 0.00392864 | protein_coding | down |
| LFNG | -1.5846164 | 8.81E-18 | protein_coding | down |
| HIST1H3B | -1.5914306 | 0.00369025 | protein_coding | down |
| HIST1H2BJ | -1.5919424 | 9.19E-16 | protein_coding | down |
| PI15 | -1.5948916 | 0.00032503 | protein_coding | down |
| SNCG | -1.5962599 | 4.22E-07 | protein_coding | down |
| CD36 | -1.5971845 | 9.40E-06 | protein_coding | down |
| NAALAD2 | -1.598206 | 1.83E-09 | protein_coding | down |
| CAVIN2 | -1.6035463 | 7.64E-15 | protein_coding | down |
| HS3ST6 | -1.6044826 | 0.00018482 | protein_coding | down |
| FNBP1L | -1.6099213 | 5.63E-16 | protein_coding | down |
| POTEM | -1.6102339 | 9.81E-05 | protein_coding | down |
| GNG2 | -1.6133997 | 2.63E-05 | protein_coding | down |
| KRT14 | -1.6162575 | 9.05E-16 | protein_coding | down |
| TGFBR1 | -1.6249484 | 1.65E-24 | protein_coding | down |
| LSP1 | -1.6309133 | 4.06E-11 | protein_coding | down |
| AKAP5 | -1.6335089 | 3.51E-09 | protein_coding | down |
| HS6ST2 | -1.6431091 | 5.72E-13 | protein_coding | down |
| PLCE1 | -1.6512871 | 2.96E-10 | protein_coding | down |
| UNC80 | -1.6615568 | 0.0005745 | protein_coding | down |
| ANXA10 | -1.6631847 | 1.71E-25 | protein_coding | down |
| LDHA | -1.6681517 | 1.32E-15 | protein_coding | down |
| NPFFR1 | -1.6693442 | 3.33E-14 | protein_coding | down |
| SEMA3B | -1.6750774 | 1.98E-21 | protein_coding | down |
| FAM110D | -1.6760655 | 8.06E-05 | protein_coding | down |
| SEMA3G | -1.6779555 | 2.02E-06 | protein_coding | down |
| MAP2K4 | -1.6823552 | 1.62E-22 | protein_coding | down |
| HIST1H2AC | -1.6932697 | 7.92E-20 | protein_coding | down |
| NEK7 | -1.7068412 | 3.41E-22 | protein_coding | down |
| MATN2 | -1.7073275 | 4.51E-12 | protein_coding | down |
| SCOC | -1.7076348 | 9.06E-28 | protein_coding | down |
| ANXA8 | -1.7101415 | 3.52E-09 | protein_coding | down |
| POLR3K | -1.7175527 | 1.48E-21 | protein_coding | down |
| FGFR3 | -1.7260763 | 7.85E-14 | protein_coding | down |
| ALOX5AP | -1.7291375 | 0.00492129 | protein_coding | down |
| MATN3 | -1.7304255 | 2.29E-14 | protein_coding | down |
| FIBIN | -1.7326115 | 0.00519018 | protein_coding | down |
| TREH | -1.7423219 | 1.93E-06 | protein_coding | down |
| SESN3 | -1.7436491 | 1.19E-24 | protein_coding | down |
| GPM6A | -1.744564 | 0.00123595 | protein_coding | down |
| METTL7A | -1.7493556 | 1.17E-07 | protein_coding | down |
| LGI3 | -1.7504405 | 7.36E-05 | protein_coding | down |
| PLCB4 | -1.7614541 | 0.00033309 | protein_coding | down |
| LBH | -1.7676701 | 3.66E-14 | protein_coding | down |
| GRIN2B | -1.7707779 | 8.13E-11 | protein_coding | down |
| HMCN2 | -1.7720029 | 0.00012733 | protein_coding | down |
| LHX9 | -1.8029908 | 4.30E-06 | protein_coding | down |
| THBD | -1.8083854 | 0.00757392 | protein_coding | down |
| HIST1H1E | -1.8119143 | 0.00137916 | protein_coding | down |
| WNT7B | -1.8122486 | 1.61E-15 | protein_coding | down |
| SHE | -1.8194915 | 1.84E-06 | protein_coding | down |
| TGFB2 | -1.8329277 | 2.14E-26 | protein_coding | down |
| TNNC1 | -1.8397966 | 3.12E-12 | protein_coding | down |
| STMN1 | -1.8403832 | 7.89E-35 | protein_coding | down |
| CENPQ | -1.8432419 | 1.52E-19 | protein_coding | down |
| ZNF442 | -1.8461924 | 0.00114818 | protein_coding | down |
| ISLR | -1.8473971 | 1.78E-05 | protein_coding | down |
| ARSI | -1.8503681 | 1.56E-29 | protein_coding | down |
| CREB3L1 | -1.8506321 | 1.52E-25 | protein_coding | down |
| RPL3L | -1.861622 | 0.0051932 | protein_coding | down |
| TBX10 | -1.8852777 | 1.11E-14 | protein_coding | down |
| NEBL | -1.8963759 | 2.51E-11 | protein_coding | down |
| FAM198A | -1.9002555 | 0.00564755 | protein_coding | down |
| RBP4 | -1.9165961 | 5.52E-06 | protein_coding | down |
| HMGCS2 | -1.9179338 | 1.50E-13 | protein_coding | down |
| TBX5 | -1.9230073 | 0.00143333 | protein_coding | down |
| IGFL2 | -1.9351356 | 1.94E-06 | protein_coding | down |
| STRA6 | -1.9403124 | 2.26E-19 | protein_coding | down |
| GRAP2 | -1.9655759 | 0.00267752 | protein_coding | down |
| CD200 | -1.9655854 | 6.57E-06 | protein_coding | down |
| CLIC3 | -1.9657296 | 1.02E-11 | protein_coding | down |
| RNASE10 | -1.9657972 | 2.10E-06 | protein_coding | down |
| NES | -1.9748641 | 7.93E-35 | protein_coding | down |
| HIST1H2AI | -1.9893468 | 3.96E-05 | protein_coding | down |
| MMP7 | -1.9926548 | 2.42E-10 | protein_coding | down |
| SBSPON | -1.9963945 | 2.69E-08 | protein_coding | down |
| CAPSL | -1.9974378 | 0.00089563 | protein_coding | down |
| C6orf223 | -1.9975456 | 0.00640378 | protein_coding | down |
| HR | -2.0017604 | 1.48E-33 | protein_coding | down |
| PADI4 | -2.0065004 | 0.00126681 | protein_coding | down |
| SVEP1 | -2.0155787 | 0.00355673 | protein_coding | down |
| CRISPLD1 | -2.0158762 | 1.05E-23 | protein_coding | down |
| SLC12A8 | -2.02088 | 1.32E-20 | protein_coding | down |
| TMEM255A | -2.0340638 | 6.14E-11 | protein_coding | down |
| FGF21 | -2.0433399 | 4.45E-07 | protein_coding | down |
| HIST1H2BC | -2.0514782 | 1.53E-24 | protein_coding | down |
| DENND2A | -2.0586607 | 0.00041713 | protein_coding | down |
| AQP1 | -2.0638028 | 2.23E-13 | protein_coding | down |
| KLHL26 | -2.0974482 | 0.00331873 | protein_coding | down |
| SOX18 | -2.1472799 | 1.53E-11 | protein_coding | down |
| C9orf84 | -2.1671302 | 3.71E-10 | protein_coding | down |
| NTRK3 | -2.1827258 | 0.00033591 | protein_coding | down |
| ALPP | -2.1851629 | 3.08E-09 | protein_coding | down |
| DNAH3 | -2.1858712 | 0.00120209 | protein_coding | down |
| ACSBG1 | -2.1977362 | 0.00021254 | protein_coding | down |
| CCDC33 | -2.2051175 | 0.00196044 | protein_coding | down |
| ACKR1 | -2.241467 | 0.00067335 | protein_coding | down |
| EXOC3L2 | -2.2442358 | 2.75E-12 | protein_coding | down |
| AC051649.2 | -2.2538556 | 5.85E-05 | protein_coding | down |
| MSLNL | -2.26868 | 0.00291276 | protein_coding | down |
| TNS4 | -2.2709127 | 3.63E-25 | protein_coding | down |
| EFHD1 | -2.2879033 | 3.52E-06 | protein_coding | down |
| CD34 | -2.2931754 | 1.43E-06 | protein_coding | down |
| CLVS2 | -2.3012964 | 3.28E-06 | protein_coding | down |
| ANKRD2 | -2.3131928 | 0.00054034 | protein_coding | down |
| C3 | -2.3200961 | 2.55E-32 | protein_coding | down |
| HSPB2 | -2.3457291 | 0.00164131 | protein_coding | down |
| ALDH1A1 | -2.3485633 | 0.00137484 | protein_coding | down |
| U2AF1 | -2.386353 | 0.00787695 | protein_coding | down |
| FRZB | -2.4044394 | 0.00877926 | protein_coding | down |
| ATP2B3 | -2.4097157 | 0.00080775 | protein_coding | down |
| JAKMIP2 | -2.4201124 | 2.84E-12 | protein_coding | down |
| ACTBL2 | -2.4585752 | 1.07E-08 | protein_coding | down |
| CRYAB | -2.4633724 | 2.39E-49 | protein_coding | down |
| MEOX1 | -2.4719138 | 0.00336513 | protein_coding | down |
| HRCT1 | -2.4722747 | 9.41E-22 | protein_coding | down |
| FOXS1 | -2.5021347 | 1.67E-10 | protein_coding | down |
| MAP2K6 | -2.5084836 | 6.96E-08 | protein_coding | down |
| FBN3 | -2.5301825 | 0.00103057 | protein_coding | down |
| CCL2 | -2.5423274 | 1.71E-05 | protein_coding | down |
| GPER1 | -2.5493455 | 1.20E-27 | protein_coding | down |
| KRT5 | -2.6234257 | 1.31E-07 | protein_coding | down |
| CLDN7 | -2.6270204 | 0.00229441 | protein_coding | down |
| ACKR3 | -2.654809 | 5.79E-31 | protein_coding | down |
| LIPG | -2.6994083 | 0.0044348 | protein_coding | down |
| KRT71 | -2.7075527 | 0.00013252 | protein_coding | down |
| PPP2R2B | -2.7250117 | 4.10E-08 | protein_coding | down |
| PTGER3 | -2.7607733 | 0.00761003 | protein_coding | down |
| RARRES2 | -2.7970736 | 3.10E-08 | protein_coding | down |
| RSPO3 | -2.8497524 | 4.38E-11 | protein_coding | down |
| ABCB5 | -2.8779734 | 1.35E-06 | protein_coding | down |
| SCNN1A | -2.955729 | 0.00340861 | protein_coding | down |
| IP6K3 | -2.9730749 | 0.00287145 | protein_coding | down |
| SPAAR | -3.0783835 | 9.57E-10 | protein_coding | down |
| HIST1H4D | -3.141028 | 0.0006623 | protein_coding | down |
| LRRC15 | -3.1815505 | 2.30E-16 | protein_coding | down |
| GPR20 | -3.3020586 | 0.0028659 | protein_coding | down |
| P2RX6 | -3.3674134 | 5.45E-05 | protein_coding | down |
| ATP10A | -3.3755822 | 5.26E-05 | protein_coding | down |
| TRIM29 | -3.4157673 | 3.50E-15 | protein_coding | down |
| EDN2 | -3.4166646 | 1.06E-45 | protein_coding | down |
| TNNT2 | -3.438813 | 6.37E-28 | protein_coding | down |
| HOXD11 | -3.6454151 | 1.14E-05 | protein_coding | down |
| OLR1 | -3.7027252 | 6.84E-06 | protein_coding | down |
| CALHM3 | -3.7781125 | 0.00065621 | protein_coding | down |
| C9orf135 | -3.8414435 | 0.00051975 | protein_coding | down |
| IGFL3 | -4.4004434 | 3.74E-09 | protein_coding | down |
| HOXD10 | -4.4064682 | 5.57E-11 | protein_coding | down |
| MYOCD | -4.6353002 | 0.00017623 | protein_coding | down |
| AOAH | -4.873202 | 0.00010043 | protein_coding | down |

| **Table S4. Antibodies for western blot and IHC used in this study** | | |
| --- | --- | --- |
| **Name** | **Company** | **Catalog Number** |
| GAPDH | bioworld | AP0066 |
| CDK4 | abcam | ab199728 |
| CyclinD | abcam | ab16663 |
| Akt | proteintech | 10176-2-AP |
| P-Akt | SANTA CRUZ | sc-33437 |
| Caspase3 | Cell Signaling | #9662 |
| Cleaved-Caspase3 | Affinity | AF7022 |
| HuR | abcam | ab200342 |
| SOX18 | Affinity | DF8018 |
| P16 | abcam | ab211542 |
| P27 | abcam | ab92741 |
| CDK6 | Cell Signaling | #3136 |
| Ki67 | abcam | ab16667 |
| Goat Anti-Rabbit | proteintech | SA00001-2 |
| Goat Anti-Mouse | proteintech | SA00001-1 |

| **Table S5. Clinicopathological characteristics of osteosarcoma patients** | | | | | |
| --- | --- | --- | --- | --- | --- |
| **Case** | **Gender** | **Age (Y)** | **Tumor size (cm)** | **Clinical stage** | **Distant metastasis** |
| 1 | Male | 27 | 25*22*11 | Ⅳ | Yes |
| 2 | Male | 16 | 6*7*3 | ⅡA | No |
| 3 | Male | 13 | 3*3*2 | Ⅰ | No |
| 4 | Male | 19 | 11*9*7 | Ⅲ | No |
| 5 | Male | 21 | 9*5*3 | ⅡA | No |
| 6 | Male | 25 | 15*13*6 | Ⅲ | No |


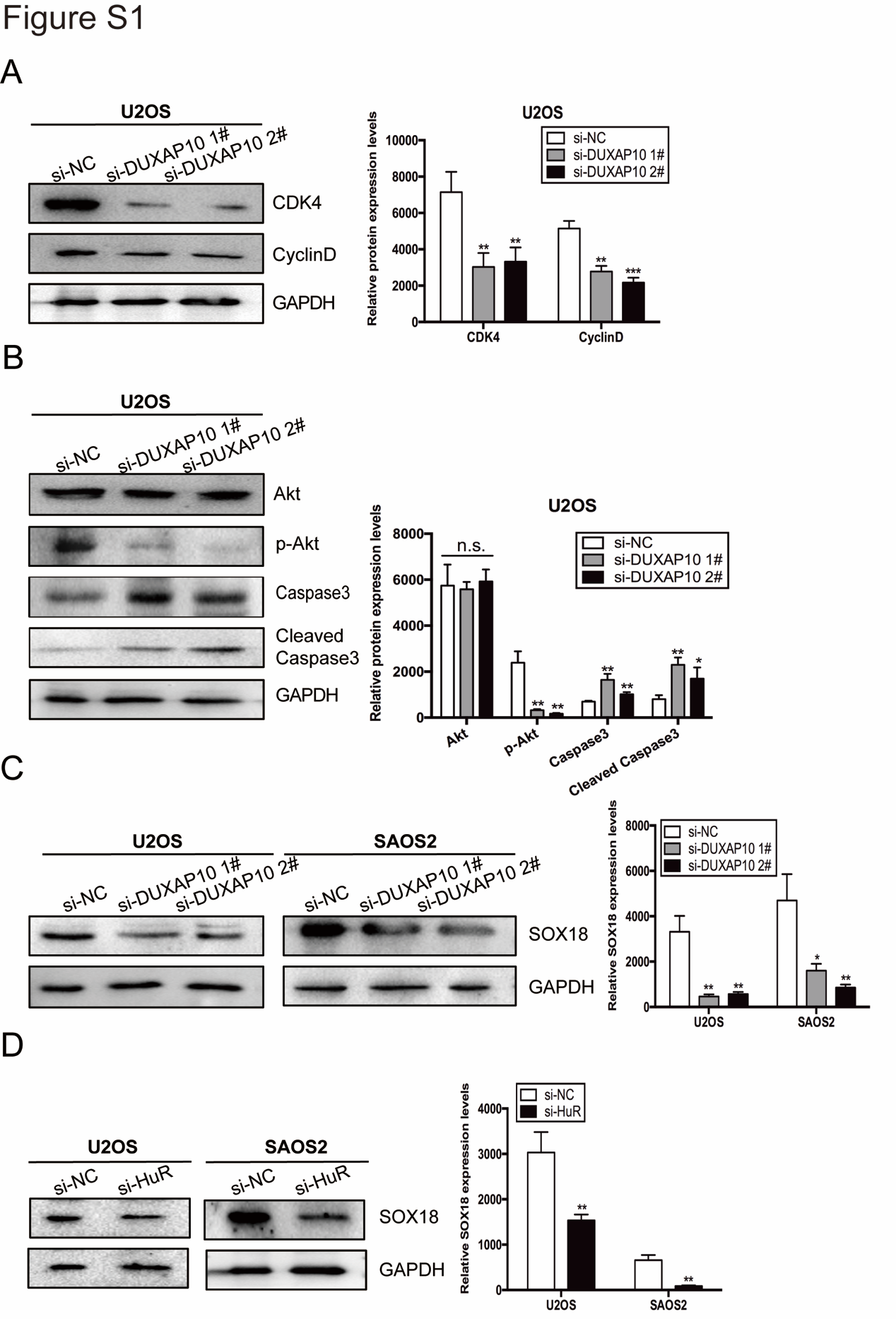


**Fig. S1 Quantitative analysis of western blot results. A&B** Western blot assay was used to detect the change of cell cycle-regulated and apoptosis-regulated protein expression in DUXAP10 knockdown U2OS cells. **C** Western blot assay was used to detect the change of SOX18 protein expression in DUXAP10 knockdown U2OS and SAOS2 cells. **D** Western blot assay was used to detect the change of SOX18 protein expression in HuR knockdown U2OS and SAOS2 cells. Data are the means ± SEM of three experiments. *P < 0.05, **P < 0.01, ***P < 0.001.


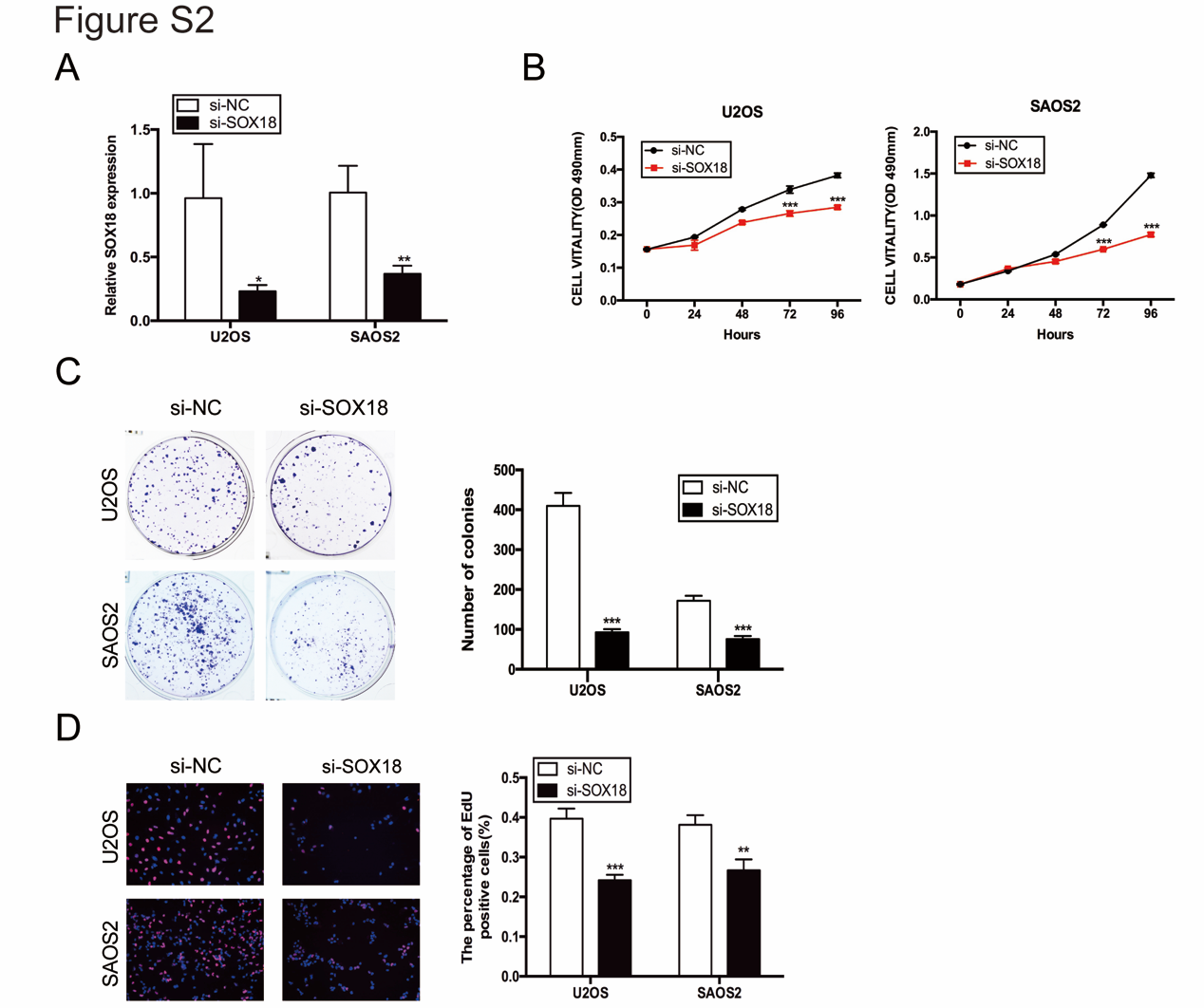


**Fig. S2 SOX18 promotes OS cell proliferation in vitro. A** Relative SOX18 expression in SOX18 knockdown U2OS and SAOS2 cells. **B** MTT assay was used to detect the effect of SOX18 on OS cell proliferation activity. **C** Colony formation assay was used to detect the effect of SOX18 on OS cell plate colony formation ability. **D** EdU assay was used to detect the effect of SOX18 on OS cell proliferation activity. Data are the means ± SEM of three experiments. *P < 0.05, **P < 0.01, ***P < 0.001.


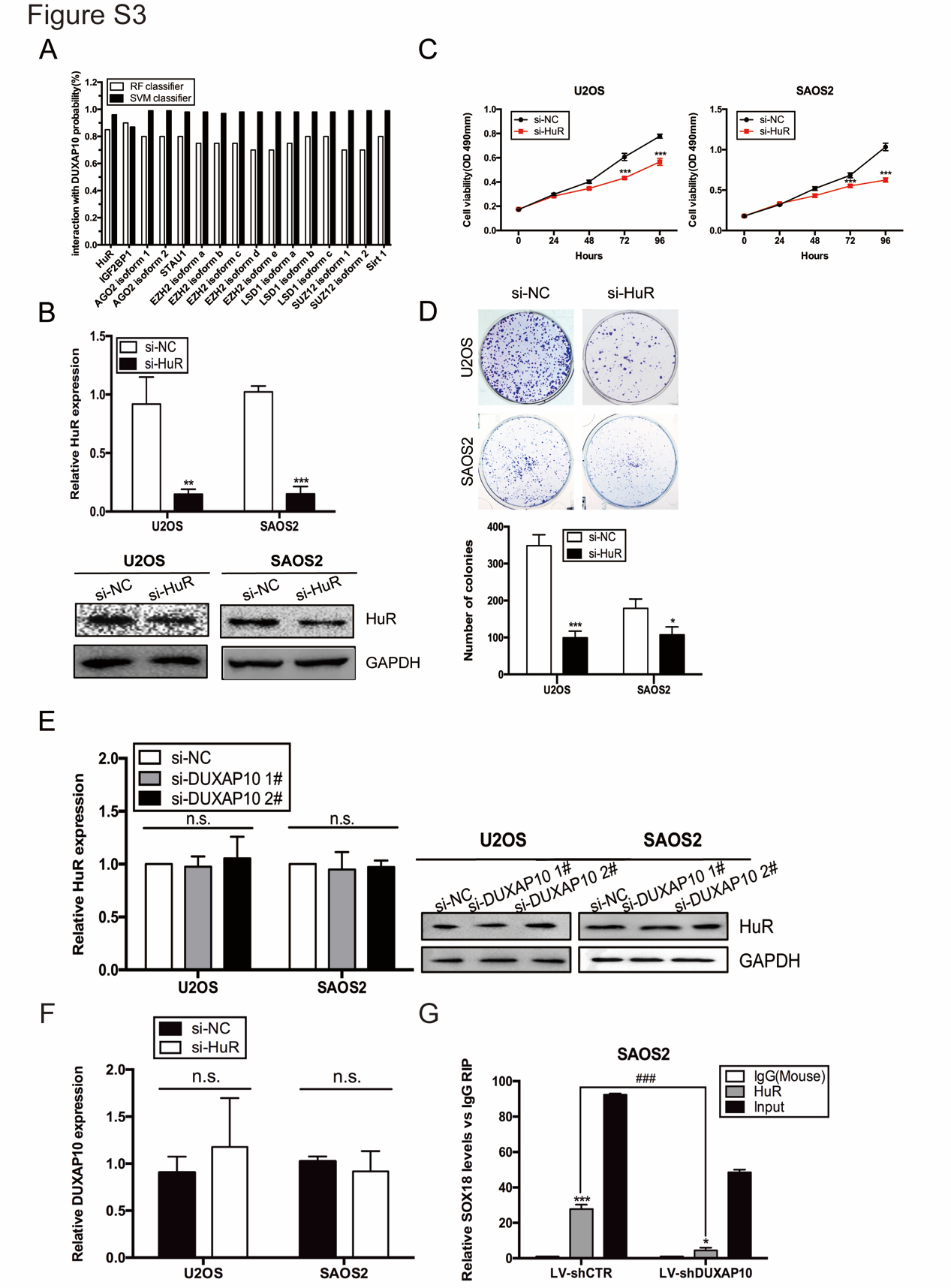


**Fig. S3 HuR promotes OS cell proliferation in vitro. A** Bioinformatics was used to detect the binding ability of RNA binding protein to DUXAP10 (http://pridb.gdcb.iastate.edu/RPISeq). **B** qRT-PCR and Western blot assays were used to detect the expression of HuR in HuR knockdown U2OS and SAOS2 cells. **C** MTT assay was used to detect the effect of HuR on OS cell proliferation activity. **D** Colony formation assay was used to detect the effect of HuR on OS cell plate colony formation ability. **E** qRT-PCR and Western blot assays were used to detect the effect of DUXAP10 knockdown on HuR expression in U2OS and SAOS2 cells. **F** Relative DUXAP10 expression in HuR knockdown U2OS and SAOS2 cells. **G** RIP assay was used to detect the degree of binding of HuR to SOX18 in LV-shCTR and LV-shDUXAP10 cells. Data are the means ± SEM of three experiments. *P < 0.05, **P < 0.01, ***P < 0.001.


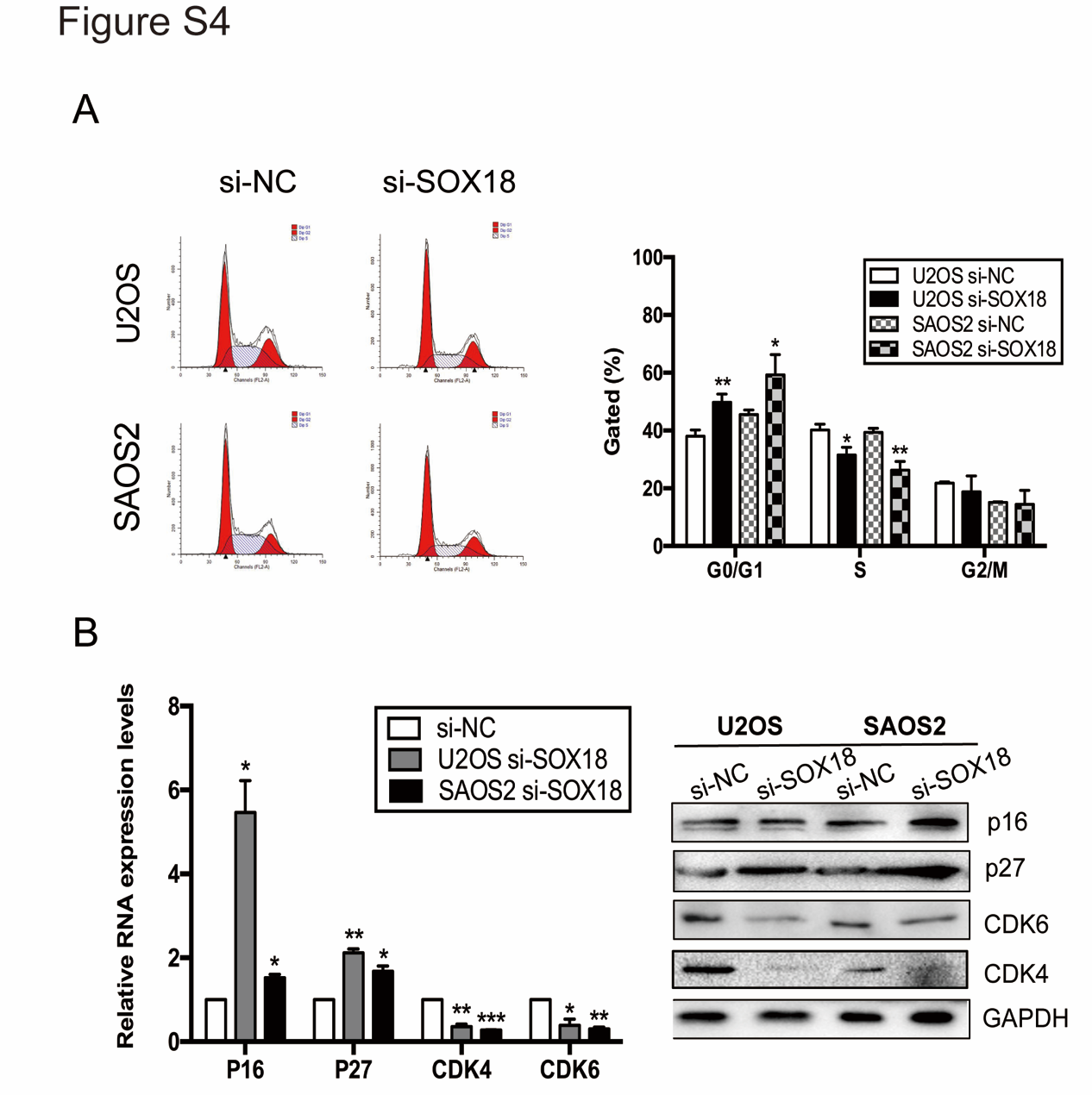


**Fig. S4 SOX18 can regulate OS cell cycle. A** Flow cytometry assay was used to detect the effect of SOX18 knockdown on OS cells’ cell-cycle. **B** qRT-PCR and Western blot assays were used to detect the expression of cell cycle related genes at both mRNA and protein levels in SOX18 knockdown U2OS and SAOS2 cells. Data are the means ± SEM of three experiments. *P < 0.05, **P < 0.01, ***P < 0.001.


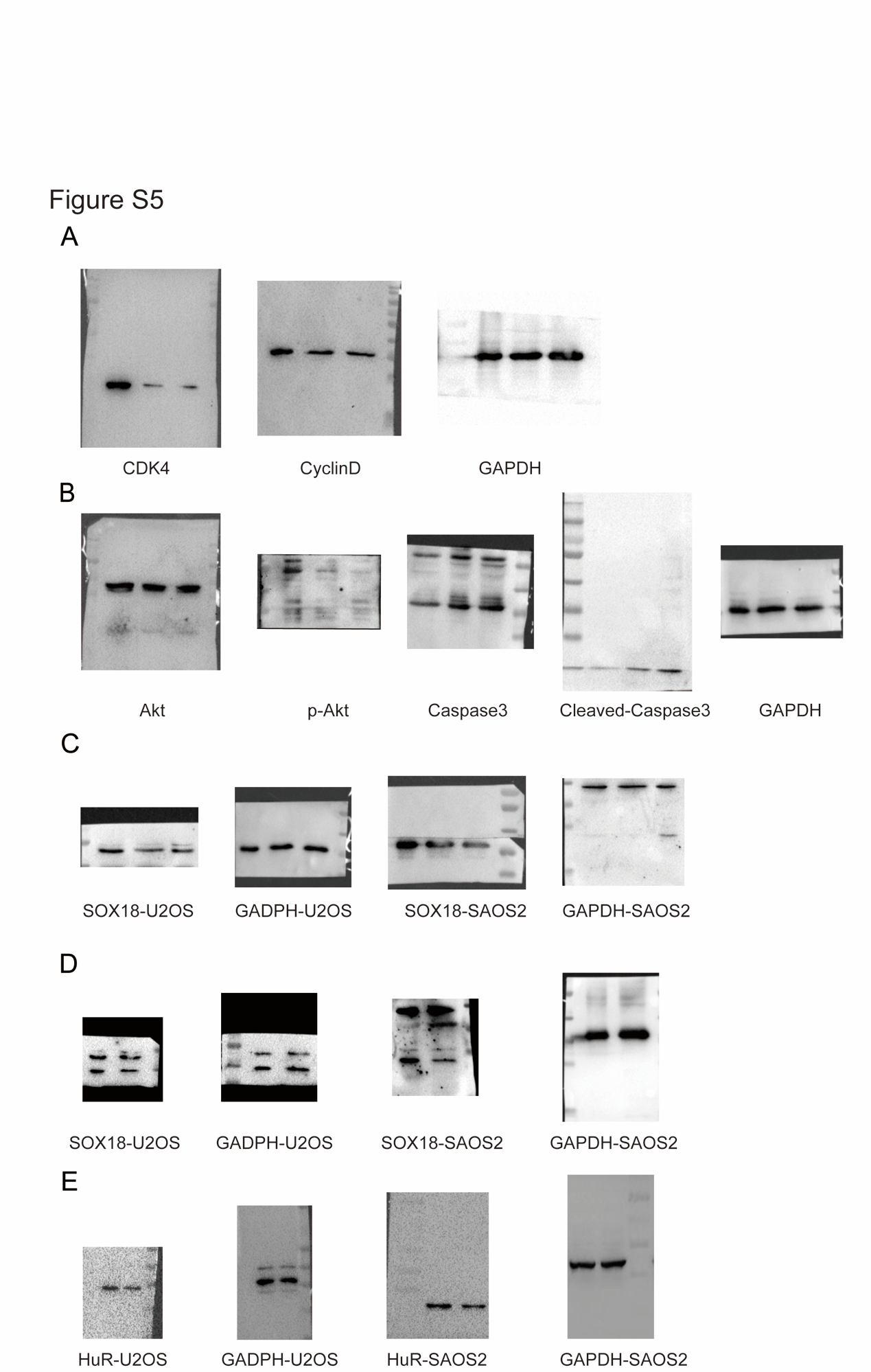


**Fig. S5 The original uncropped immunoblot images. A** The original uncropped immunoblot images used in Fig. 3C. **B T**he original uncropped immunoblot images used in Fig. 3D. **C T**he original uncropped immunoblot images used in Fig. 5C. **D T**he original uncropped immunoblot images used in Fig. 6C. **E T**he original uncropped immunoblot images used in Fig. S3B.
